# Mast Cells Expedite Control of Pulmonary Murine Cytomegalovirus Infection by Enhancing the Recruitment of Protective CD8 T Cells to the Lungs

**DOI:** 10.1371/journal.ppat.1004100

**Published:** 2014-04-24

**Authors:** Stefan Ebert, Marc Becker, Niels A. W. Lemmermann, Julia K. Büttner, Anastasija Michel, Christian Taube, Jürgen Podlech, Verena Böhm, Kirsten Freitag, Doris Thomas, Rafaela Holtappels, Matthias J. Reddehase, Michael Stassen

**Affiliations:** 1 Institute for Virology and Research Center for Immunology (FZI), University Medical Center of the Johannes Gutenberg University, Mainz, Germany; 2 Institute for Immunology and Research Center for Immunology (FZI), University Medical Center of the Johannes Gutenberg University, Mainz, Germany; 3 Department of Pulmonology, Leiden University Medical Center, Leiden, The Netherlands; University of Southern California, United States of America

## Abstract

The lungs are a noted predilection site of acute, latent, and reactivated cytomegalovirus (CMV) infections. Interstitial pneumonia is the most dreaded manifestation of CMV disease in the immunocompromised host, whereas in the immunocompetent host lung-infiltrating CD8 T cells confine the infection in nodular inflammatory foci and prevent viral pathology. By using murine CMV infection as a model, we provide evidence for a critical role of mast cells (MC) in the recruitment of protective CD8 T cells to the lungs. Systemic infection triggered degranulation selectively in infected MC. The viral activation of MC was associated with a wave of CC chemokine ligand 5 (CCL5) in the serum of C57BL/6 mice that was MC-derived as verified by infection of MC-deficient *Kit^W-sh/W-sh^* “sash” mutants. In these mutants, CD8 T cells were recruited less efficiently to the lungs, correlating with enhanced viral replication and delayed virus clearance. A causative role for MC was verified by MC reconstitution of “sash” mice restoring both, efficient CD8 T-cell recruitment and infection control. These results reveal a novel crosstalk axis between innate and adaptive immune defense against CMV, and identify MC as a hitherto unconsidered player in the immune surveillance at a relevant site of CMV disease.

## Author Summary

Being strategically located beneath endothelial and epithelial surfaces, mast cells (MC) serve as sentinels for invading pathogens at host-environment boundaries as part of the innate defense against infection. Host genetic resistance against cytomegaloviruses (CMV) is largely determined by the innate immune response, but an implication of MC in the adaptive immune defense against CMV has not been considered so far and is almost impossible to address in human infection. Using murine CMV as a model that in the past has already pioneered the discovery of fundamental principles in CMV-host interactions, our data reveal MC as central part of a novel crosstalk-axis between the innate and adaptive immune response to CMV. We found that upon host infection MC become rapidly activated and promote the recruitment of protective CD8 T cells to the lungs, a noted critical site of CMV pathogenesis in humans as well as in the mouse model. Enhanced tissue infiltration of CD8 T cells results in a reduced peak viral load and a faster clearance of productive infection. Realizing the importance of MC in the control of pulmonary CMV infection may help to develop new strategies for preventing CMV pneumonia by MC supplementation in recipients of hematopoietic cell transplantation.

## Introduction

CMVs are dsDNA viruses of the beta-herpesvirus subfamily. Human CMV infection is still of major concern in clinical practice, in particular in immunocompromised patients in whom latent virus can reactivate. Specifically, symptomatic CMV infection after hematopoietic cell transplantation (HCT) is a serious complication that can cause engraftment failure and multiple organ disease associated with significant morbidity and mortality, both in clinical HCT (for reviews, see [1], [2]) as well as in the murine CMV (mCMV) experimental model ([3], [4] reviewed in [5]). Interstitial pneumonia is the most critical clinical manifestation of CMV disease in patients post-HCT ([6], [7], [8] for a recent review, see [2]). In accordance with these clinical findings, the murine model in immunocompromised host and experimental HCT settings has identified the lungs as a predilection site of CMV in acute infection [9], [10], [11] as well as in CMV latency and reactivation [11], [12], [13], [14]. Antiviral CD8 T cells are noted as the main direct effector cells to control acute CMV infection following HCT and, accordingly, their timely reconstitution is indispensable for survival ([9], [11], [15] reviewed in [5]).

We have previously shown in different models of mCMV infection, including HCT and adoptive CD8 T-cell transfer models as well as in the immunocompetent host, that control of acute pulmonary mCMV infection [11], [16], but also of infection at other sites, such as in liver parenchyma [17], [18], correlates with the formation of nodular inflammatory foci (NIF). NIF are structures in which effector CD8 T cells selectively accumulate at infected tissue cells, thereby preventing further virus spread and ultimately clearing productive tissue infection. After depletion of CD8 T cells, CD4 T cells still infiltrate tissues in a scattered distribution but do not form NIF and, accordingly, fail to control the infection ([11], reviewed in [5]). Formation of NIF was more recently also revealed in the lungs of mCMV-infected neonatal mice by 2-photon microscopy showing co-localization of infected, highly mCMV-susceptible pneumocytes with myeloid lineage-derived antigen-presenting cells (APC) and T cells, followed by their disruption and clearance of the infection [19]. It was proposed that T-cell priming/stimulation and proliferation also take place in NIF [19]. In this context, importantly, NIF formation was found to depend on the expression of the cognate antigen by the infected tissue cells [20]. Specifically, after transfer of effector CD8 T cells specific for the IE1 epitope YPHFMPTNL of mCMV [21] into immunocompromised mice, protective NIF were formed in recipient mice infected with wildtype (WT) or revertant viruses encoding the authentic IE1 peptide but not in recipient mice infected with an “antigenicity loss” mutant virus coding for the IE1-Ala analog YPHFMPTNA [22] that is not generated as a peptide by antigen processing in the first place and, in addition, replacement of C-terminal residues of antigenic peptides with alanine strongly reduces the binding affinity to the presenting MHC class I molecule ([23], [24], reviewed in [25]).

Previous work has identified endothelial cells (EC), interstitial fibroblasts, and pneumocytes of the alveolar epithelium as target cells of acute mCMV infection in the lungs [9], [18], [26]. Thus, in order to form NIF and exert a protective function within lung interstitium and parenchyma, antiviral CD8 T cells must leave the vascular compartment by transit through vascular endothelium into extravascular compartments. As shown previously by Galkina and colleagues [27], even under conditions of a noninflamed pulmonary interstitium, effector CD8 T cells preferentially egress from the pulmonary vascular compartment into pulmonary interstitium in a two-step process involving leucocyte function antigen-1 (LFA-1)-dependent retention within lung vasculature followed by extravasation driven in part by CC-chemokine ligand 5 (CCL5), also known as RANTES [28]. MC-derived CC-chemokine receptor ligands, including CCL5, were also reported to induce migration of a subset of human natural killer T (NKT) cells *in vitro* in chemotaxis assays [29]. As shown by toll-like receptor (TLR)3 ligation with poly I:C, activated mast cells secrete CCL5 and can recruit effector CD8 T cells from lymphoid tissues to non-lymphoid compartments, into the peritoneal cavity in the reported case [30].

MC reside in all vascularized tissues and can be found primarily in close proximity to vessels and nerves as well as beneath endothelial and epithelial surfaces. The ability of MC to act as the immune system's “loaded gun” [31] is based on the rapid release of potent proinflammatory and antimicrobial mediators. Some of these mediators are already stored in secretory granules and their release on demand relies on the expression of a variety of receptors that allow the stimulation of MC independently of the canonical MC receptor IgE. The receptor repertoire includes numerous pattern recognition receptors encompassing most TLR, including TLR3 and TLR9 [32], [33], [34].

Based on MC being noted producers of CCL5, we pursued the idea that MC might possibly play a role in the recruitment of protective antiviral CD8 T cells to mCMV-infected lungs. To the best of our knowledge, there exist no functional data on MC activation by CMVs *in vivo* not to mention data on a role MC might play in the immune control of CMV infections *in vivo*. To investigate a potential implication of MC in T-cell recruitment with a focus on pulmonary infection, we applied the experimental mCMV model for comparing the antiviral immune response in the lungs of MC-deficient *Kit^W-sh/W-sh^* (briefly *Kit^W-sh^* or “sash”) mice [35], [36], [37] and in MC-sufficient congenic WT C57BL/6 mice as well as in MC-reconstituted “sash” mutants.

Using a green fluorescent protein (GFP)-mCMV reporter virus [38], we show here that mCMV infects MC *in vivo* and triggers degranulation selectively of GFP-expressing infected MC, but not of GFP-negative uninfected MC. This finding implies a contribution of direct virus-MC interaction is required for MC degranulation, and that virally induced cytokines derived from third party target cells of infection are either not involved or not sufficient. Importantly, MC activation by mCMV induced a serum wave of MC-derived CCL5, associated with CD8 T-cell infiltration of lung tissue and only low-level virus replication. Conversely, shortage of CCL5 in the serum of infected MC-deficient “sash” mutants was associated with reduced CD8 T-cell recruitment and consequently elevated virus replication and delayed clearance of productive infection.

These findings thus identify a novel crosstalk axis between innate and adaptive immune defense in the immune surveillance of CMV infection with a translational perspective for use of MC supplementation in T cell-based immunotherapy of CMV disease.

## Results

### Lung-infiltrating CD8 T cells form NIF in lung parenchyma for controlling infection

The formation of NIF as microanatomical sites of infection confinement in tissues has originally been described for BALB/c (H-2^d^ haplotype) mice (see the Introduction), in which the infection at most sites, except persistent infection of salivary glands, is controlled primarily by CD8 T cells ([39]; for a review, see [40]). Unlike BALB/c mice, which do not express the activatory natural killer (NK) cell receptor Ly49H, early control of mCMV replication in mice of C57BL/6 (H-2^b^ haplotype) background, particularly in the spleen, is mediated by NK cells through ligation of Ly49H with a virally encoded major histocompatibility (MHC) class I-like ligand (MHC-Iv) m157 [41], [42]. These findings are sometimes misinterpreted to mean that CD8 T cells play generally no role in C57BL/6 infection models. However, even though the strong early m157-Ly49H activated NK-cell response inhibits an early CD8 T-cell response [43], virus replication at times later than day 3–4 after intravenous infection of immunocompetent C57Bl/6 mice is controlled also by CD8 T cells, even in the spleen [43].

**Figure 1:**
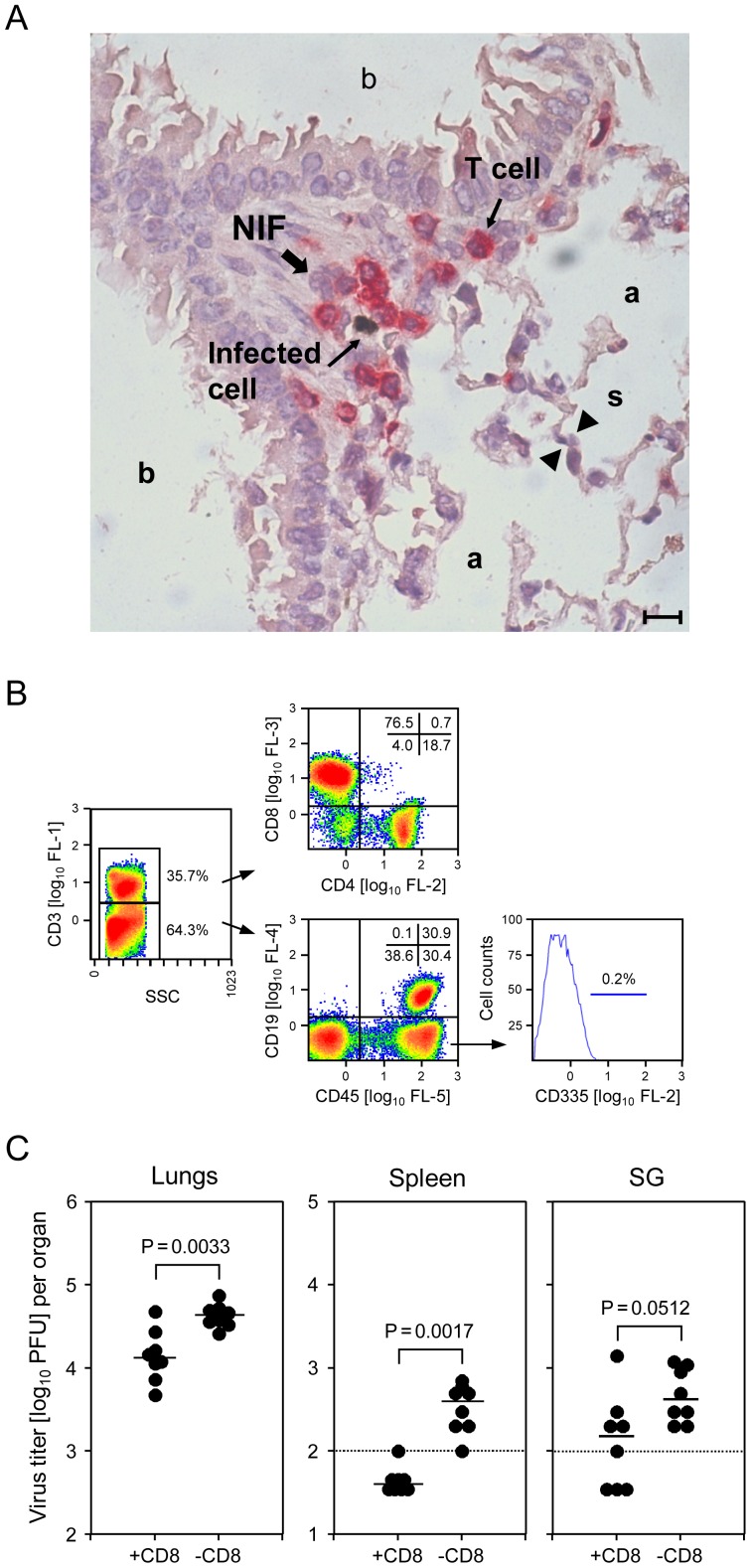
CD8 T cells localizing to nodular inflammatory foci (NIF) control pulmonary infection. (A) Infected cells in alveolar epithelium and lung-infiltrating T cells co-localize in NIF. Lung tissue sections were taken on day 6 after intravenous infection of WT C57BL/6 mice. 2-color immunohistological staining identifies infected cells by black staining of viral intranuclear IE1 protein and T cells by red staining of CD3ε. (a) pulmonary alveoli; (b) pulmonary bronchioles lined with bronchiolar epithelium; (s) alveolar septum. The bar marker represents 10 µm. (B) Cytofluorometric phenotyping of pulmonary leucocyte subpopulations revealing a high proportion of CD3^+^CD8^+^ T cells and virtual absence of CD335^+^ NK cells. The analysis was performed for a pool of 6 mice on day 6, corresponding to the immunohistological localization study. Shown are color-coded density plots with red and blue representing highest and lowest density, respectively. Percentages of main interest are indicated for gated areas and quadrants. (SSC) side scatter in the live gate; (FL) fluorescence channel and fluorescence intensity. (C) Infection of lungs and spleen is controlled by CD8 T cells. C57BL/6 mice were depleted of CD8 T cells on the day before infection. Virus titers in homogenates of the indicated organs were determined on day 6 post-infection. Dots represent virus titers in individual mice revealing the sample sizes and ranges, with the median values indicated. The dotted lines indicate the detection limits.

To directly address this issue with special emphasis on the immune control of virus replication in the lungs at a time beyond the early-stage NK-cell response, we tested the contribution of lung-infiltrating T cells to the later-stage antiviral control on day 6 after intravenous infection of immunocompetent, adult C57BL/6 mice. Direct imaging of lung tissue by 2-color immunohistology, with staining of CD3ε on T cells in red color and viral immediate-early 1 (IE1) protein in the nuclei of infected cells in black, shows a typical NIF located in the alveolar epithelium (Fig. 1A). Obviously, red-stained T cells are not randomly distributed but accumulate selectively in a NIF and literally enwrap an infected cell, most likely a pneumocyte, to seclude it from neighboring cells for preventing virus spread. Notably, bronchiolar epithelium is spared from infection, and thus also from focal T-cell infiltration.

We were then interested in defining the subset composition of the tissue-infiltrating CD3^+^ T cells as well as of non-T cells. In the representative example shown (Fig. 1B), cytofluorometric analysis of single-cell preparations of collagenase-digested lung tissue revealed ca. 1/3 of the cells are CD3^+^ cells, amongst which CD8 T cells predominate by far, with only ca. 20% CD4 T cells and a very minor population of CD3^+^CD4^−^CD8^−^ cells supposed to comprise NKT and γ/δ T cells. Gating on CD3^−^ cells followed by analysis of CD19 expression on B cells and of the pan-leucocyte marker CD45 gave the important information that cell preparations from the lungs can contain substantial numbers of CD45^−^ cells, that is cells not belonging to hematopoietic lineages. Thus, these cells are not lung-infiltrating leucocytes but lung tissue cells, a finding that calls for a routine pre-gating on CD45^+^ cells in all further analyses of lung-infiltrating leucocyte subpopulations. Amongst the CD45^+^ cells, ca. 50% were B cells. The remaining CD3^−^CD45^+^CD19^−^ population includes myeloid lineage cells, such as macrophages and dendritic cells, as well as NK cells. Regarding the discussion on the contribution of NK cells to antiviral control in the C57BL/6 model, we were most interested in quantifying NK cells, supposed to be enriched in the CD3^−^CD45^+^CD19^−^ lung leucocyte population. Unexpectedly, we did not find a noticeable number of CD335^+^ (NKp46) NK cells [44] (Fig. 1B), while these cells represent an easily detectable, distinct population in the spleen (data not shown).

Having demonstrated that CD8 T cells predominate in the lung-infiltrating CD3^+^ population, their contribution to the control of the infection was verified by quantitating infectious virus in the lungs, and in spleen and salivary glands for comparison, dependent upon presence or absence of CD8 T cells (Fig. 1C). Depletion of CD8 T cells significantly enhanced virus replication in lungs and spleen. As expected, the impact of CD8 T-cell depletion was low for virus load in the salivary glands on day 6, as virus replication in salivary glands is known to be at that time redundantly controlled also by NK1.1^+^ NK cells, so that only combined depletion of NK1.1^+^ NK cells and CD8 T cells elevates virus titers [43]. Based on virtual absence of NK cells in the lungs (Fig. 1B), we did not predict a major influence of m157-activated Ly49H^+^ NK cells at a more advanced stage of the infection. Indeed, on day 14, virus titers in the lungs were the same for C57BL/6 mice infected intravenously with WT virus or mutant virus deleted for the m157 gene [45] (Fig. S1). By the time of analysis, productive infection was cleared in the spleen for both viruses, whereas productive infection persisted in the salivary glands on a level strongly influenced by presence or absence of NK cell activation through m157-Ly49H (Fig. S1).

Altogether, this set of data has confirmed a major role for CD8 T cells in the control of pulmonary mCMV infection in the C57BL/6 model of an immunocompetent host.

### Activated CD8 T cells leave the vascular compartment and transit into the alveolar epithelium of the lungs

By using a short intravenous pulse of fluorochrome-conjugated anti-CD8a antibody for intravascular staining of CD8 T cells [27], recent work has indicated that most effector and memory CD8 T cells recovered after perfusion from digested lung tissue, more than 90% in some contexts, actually do not localize to lung parenchyma but are retained in the narrow capillary network associated with the alveoli [46]. This method, however, is critically based on the condition that the endothelium of pulmonary capillaries is impermeable for the fluorochrome-conjugated antibody used for intravascular staining. This condition is not fulfilled for acute mCMV as well as human CMV infections, as these cytopathogenic viruses infect endothelial cells [47] and cause capillary leakiness, which, in the immunocompromised host, can develop into a “capillary leak syndrome” as one clinical manifestation of CMV disease becoming evident as petechial bleedings in skin and mucosa. In fact, work using a floxed-EGFP recombinant mCMV for infection of Tie2-Cre mice showed that essentially all virus present in lungs has recombined in endothelial cells to express EGFP prior to spread into lung parenchyma [18]. In addition, infection-associated pro-inflammatory cytokines could also have an influence on the endothelium's integrity and permeability. Specifically, activated MC release factors such as tumor necrosis factor α (TNFα), histamine, proteases, and chemokines that are known to increase vascular permeability at sites of ongoing infection [48]. These limitations must be considered whenever the otherwise elegant technique of intravascular staining is applied.

**Figure 2:**
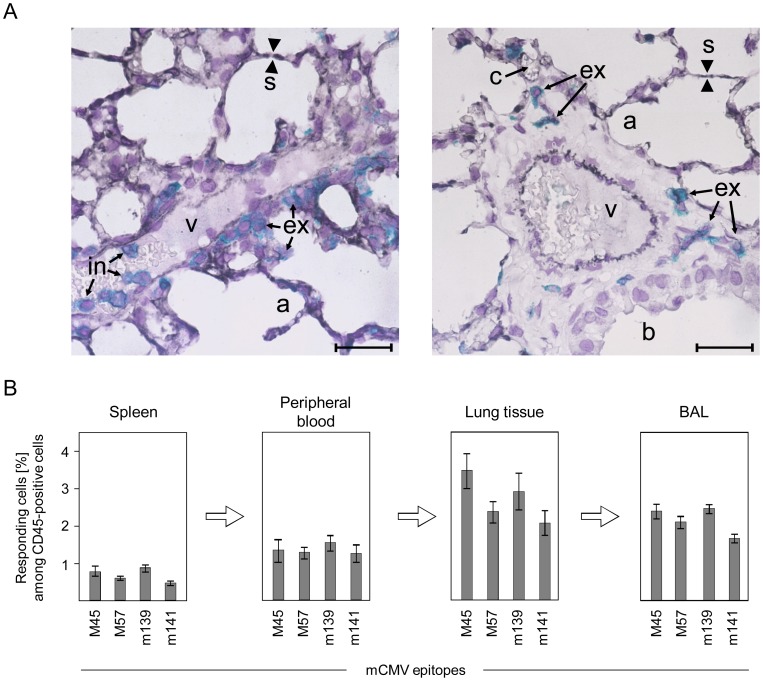
Activated virus epitope-specific CD8 T cells transmigrate from the vascular compartment into lung interstitium and alveolar epithelium. (A) Localization of CD3^+^ T cells in intravascular (in) and extravascular (ex) compartments of the lungs. Shown are 2-color immunohistological images of lung tissue sections taken on day 6 after infection of C57BL/6 mice, with endothelial cells being identified by black staining of CD31 and T cells by turquoise-green staining of CD3ε. (v) small vessel; (a) pulmonary alveoli; (b) pulmonary bronchiole lined with bronchiolar epithelium; (s) alveolar septum; (c) capillary in a thickened alveolar septum. The bar marker represents 25 µm. (B) Frequencies of viral epitope-specific, IFNγ-secreting CD8 T cells among CD45^+^ leucocytes in different compartments. CD8 T cells recovered by BAL are those that are only loosely attached to the epithelial lining of the alveoli or bronchioles and are thus definitely located outside of the lung vasculature. Shown are data from ELISpot assays using stimulator cells exogenously-loaded with saturating concentrations of antigenic peptides representing the epitopes indicated. Bars represent most probable numbers determined by intercept-free linear regression analysis and error bars represent the 95% confidence intervals.

For localizing antiviral T cells more reliably, we have therefore used a combination of direct imaging by 2-color immunohistology and an enzyme-linked immunospot (ELISpot) assay for quantitating viral epitope-specific MHC class-I H-2^b^-restricted CD8 T cells in different compartments (Fig. 2). Endothelial cells were identified by black staining of CD31, and T cells by turquoise-green staining of CD3ε (Fig. 2A). Tissue sections were selected to show small vessels in longitudinal section and cross-section (Fig. 2A, left and right panel, respectively). The left panel of Fig. 2A reveals intravascular T cells attached “head-ahead” to the vessel endothelium (in) as well as T cells that have transmigrated into the alveolar epithelium (ex). The right panel shows T cells undoubtedly localized in the lung interstitium (ex) between a vessel and the alveolar epithelium, far away from capillaries. ELISpot assays were performed to quantitate effector CD8 T cells capable of responding with IFNγ secretion to stimulation with a set of mCMV peptides characterizing the acute CD8 T-cell response to mCMV in the C57BL/6 model [49] (Fig. 2B). Relative to all CD45^+^ lymphocytes, viral epitope-specific effector CD8 T cells are enriched in the lungs compared to blood, indicating their retention in perfused lungs. This finding *per se* cannot distinguish between intravascular cells sticking to capillary endothelia and those that have transmigrated into interstitium and epithelia. Retrieval by bronchoalveolar lavage (BAL), however, definitely indicated that these cells must have emigrated from the vascular compartment to extravascular compartments. In accordance with the ELISpot data, cytofluorometric analysis of CD45^+^ leucocytes in the BAL revealed a high proportion of CD8 T cells, out of which about 2% were specific for the M57 epitope based on binding of K^b^-M57 peptide multimers to the cognate T-cell receptor (TCR) (Fig. S2).

### CMV rapidly activates mast cells *in vivo* by direct infection

Having recognized the importance of lung tissue infiltration and NIF formation for the control of pulmonary mCMV infection by CD8 T cells, along with our previous finding that NIF formation requires cognate antigen [20], we got interested in mechanisms of CD8 T-cell recruitment into infected tissue sites, with chemokines being obvious candidates. Inspired by work of Galkina and colleagues, who have implicated CCL5 in the egress of effector CD8 T cells from the vasculature to extravascular compartments of the lungs [27], along with the finding by Orinska and colleagues of CCL5 induction in poly I:C-stimulated mast cells (MC) associated with recruitment of CD8 T cells into the peritoneal cavity [30], we pursued the idea that MC might play a role in the recruitment of protective CD8 T cells to the mCMV-infected lungs, which is an aspect that has not been considered before in CMV research.

**Figure 3:**
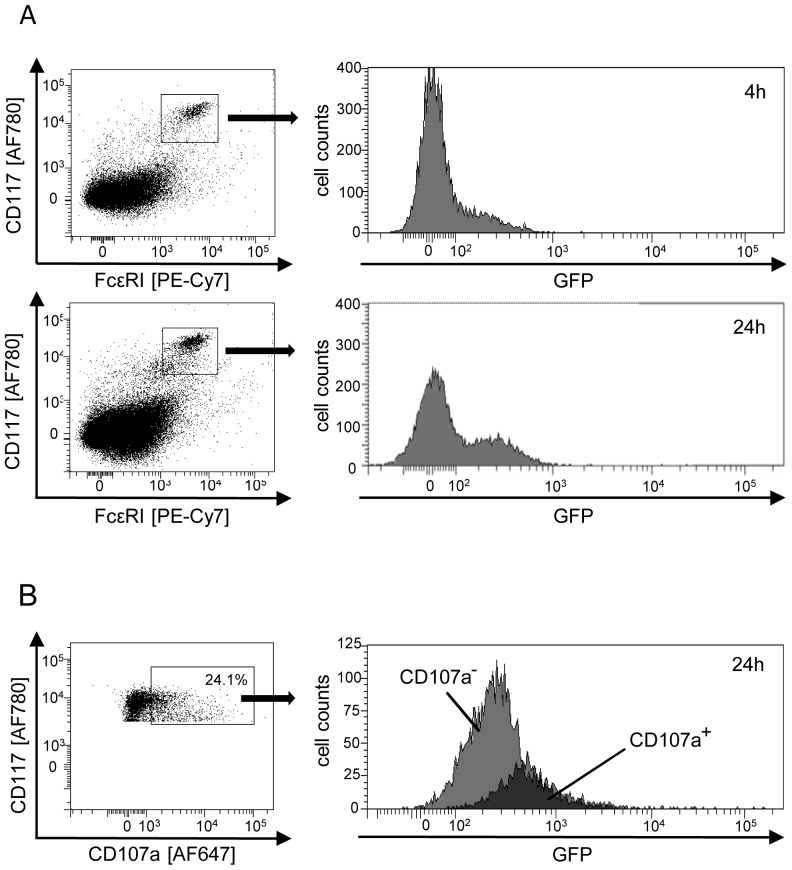
Degranulation of mast cells (MC) is triggered by direct infection *in vivo*. (A) MC are first-hit target cells of mCMV infection *in vivo*. Peritoneal exudate cells (PEC) were recovered from the peritoneal cavity at 4 h or 24 h after intraperitoneal infection with fluorescent reporter virus mCMV-GFP. This timing does not allow completion of the viral infection cycle and thus precludes virus spread to secondary target cells. In the cytofluorometric analysis, a gate was set on FcεRI^+^CD117^+^ MC for identification of infected MC expressing the reporter GFP. (B) Selective degranulation of infected, GFP^+^ MC. PEC were pre-gated for expression of FcεRI and analyzed for the expression of CD117 and the degranulation marker CD107a. GFP-expression in FcεRI^+^CD117^+^CD107a^+^ MC identifies degranulating MC as those which are infected. The black-shaded area represents cells within the gate of CD107a^+^ MC, the gray-shaded area represents the “reverse gate” of MC not or no longer expressing CD107a. Shown are the original data from one mouse as an example. Percentages of degranulating CD107a^+^ MC of n = 4 mice tested individually ranged between 9.0% and 24.1% with a mean value of 17.5% and a median value of 18.5%.

Any proposed contribution of MC to CD8 T-cell recruitment requires, in the first place, an activation of MC, be it directly by infection of MC or indirectly by CMV infection-associated ligands derived from infected other cell types but initiating signaling pathways activating MC in a paracrine fashion. Though an early report has suggested direct infection of bone marrow-derived MC (BMMC) *in vitro* [50], we were honestly not able to confirm these data with highly pure, macrophage-free populations of BMMC infected with a GFP reporter virus mCMV-GFP [38], and viral IE1 protein was undetectable in BMMC infected with different mCMV strains regardless of their expression of the recently identified gH/gL/MCK-2 glycoprotein complex that is involved in cell type-specific entry of mCMV [51] (data not shown). However, BMMC differentiated in cell culture differ in their maturation from *in vivo* MC [32] and this might affect the permissivity to infection. We therefore infected C57BL/6 mice intraperitoneally with mCMV-GFP and recovered peritoneal exudate cells (PEC) at 4 h and 24 h post-infection, times which restrict the analysis to “first hit” target cells of mCMV before progeny virions can spread. MC amongst the PEC were identified cytofluorometrically by their expression of FcεRI and CD117 (Fig. 3). While, after 4 h, GFP expression did not reveal a distinct subpopulation of infected MC, this was clearly the case after 24 h, thus demonstrating for the first time that FcεRI^+^CD117^+^ MC are “first hit” targets of mCMV infection *in vivo* (Fig. 3A).

We next addressed the decisive question of whether mCMV infection of the host activates MC and whether, in that case, MC activation is restricted to the actually mCMV-infected MC or results from paracrine activation also of uninfected MC by cytokines delivered by other infected cell types. Activation of MC was monitored by flow cytofluorometric analysis of CD107a expression on CD117^+^ MC, pre-gated for FcεRI expression. CD107a, also known as lysosomal-associated membrane protein-1 (LAMP-1), is exposed on the cell surface specifically upon degranulation of MC. A proportion of MC was found to be in the process of degranulation in response to mCMV-GFP infection *in vivo* and, most notably, degranulation was clearly restricted to GFP-expressing, infected MC (Fig. 3B). This result shows that direct infection of MC is essential for MC degranulation to occur, and excludes triggering of degranulation of uninfected MC through paracrine signals from infected MC or from infected other cell types.

### CMV infection induces a wave of mast cell-derived CC chemokine ligand 5 (CCL5/RANTES) in the serum

**Figure 4:**
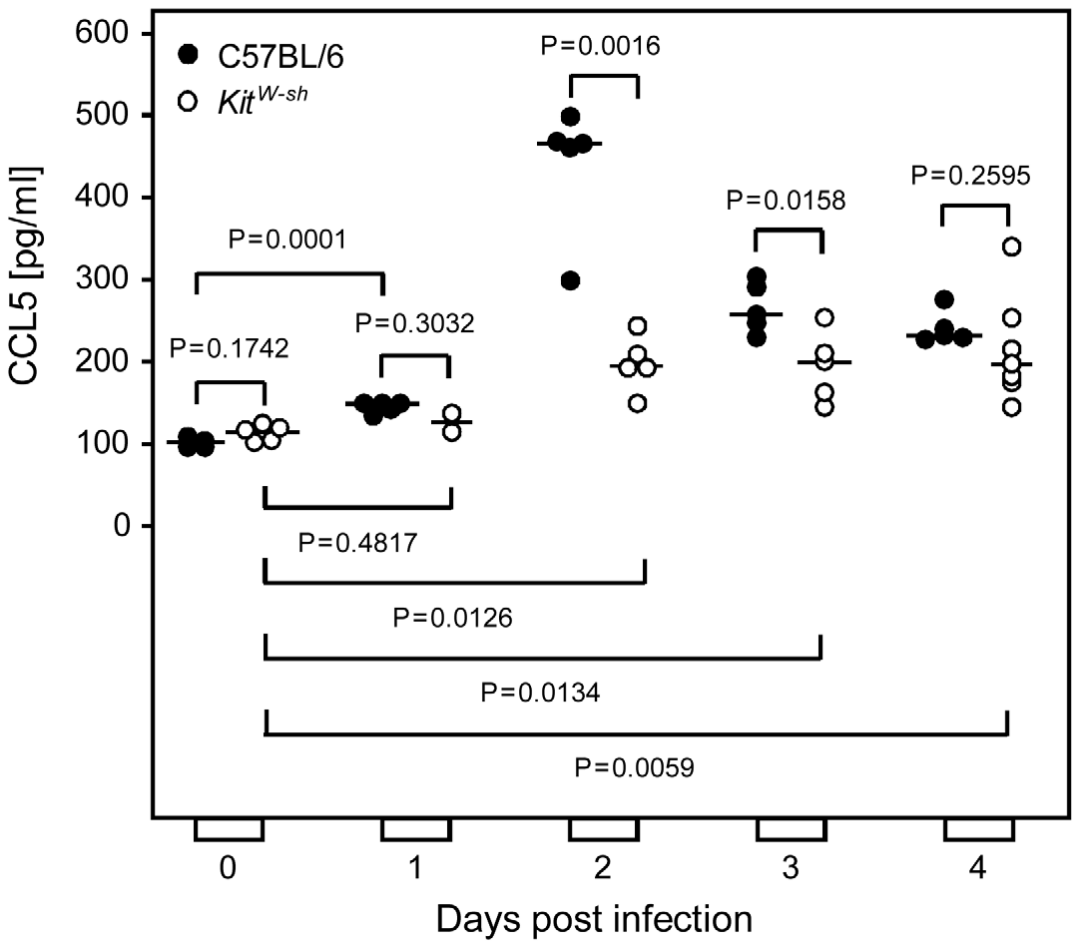
Infection induces a wave of mast cell-derived chemokine CCL5/RANTES *in vivo*. Serum levels of chemokine CCL5 were determined in a time course after intravenous infection of MC-sufficient WT C57BL/6 mice compared to congenic MC-deficient *Kit^W-sh/W-sh^* (*Kit^W-sh^*“or “sash”) mutants. Symbols represent data (mean values of duplicate serum aliquots) from individual mice revealing sample sizes and ranges, with the median values indicated. P values are given for comparisons of interest, unless the difference is obvious.

As introduced in greater detail above, CCL5 secreted by activated MC is a prime candidate for recruiting CD8 T cells to non-lymphoid organ sites of virus replication. We therefore analyzed absolute serum levels of CCL5 in immunocompetent C57BL/6 mice in response to intravenous infection in a time course (Fig. 4) and found peak levels on day 2 post-infection with a ca. 5-fold induction compared to pre-infection levels, which is quite substantial. It remained, however, the critical question if the detected CCL5 is indeed derived from MC, as proposed, or if other cell types might actually account for the CCL5 release observed. The answer was given by CCL5 levels in the serum of MC-deficient *Kit^W-sh/W-sh^* (briefly *Kit^W-sh^* or “sash”) mutants [35], [36], [37], whose genetic background is also C57BL/6 for a reliable comparison. Baseline levels of serum CCL5 pre-infection (day 0) were the same for WT C57BL/6 mice and their congenic “sash” mutants, whereas, beyond day 1, CCL5 levels were significantly lower in the MC-deficient mutant, both statistically as well as in biologically relevant quantity, until the difference was leveled on day 4, though at values still significantly above the pre-infection levels. Notably, in “sash” mutants, post-infection levels were also slightly above the pre-infection levels, ca. 2-fold between days 2 and 4. This indicates some CCL5 might come from other cell types and account for some CD8 T-cell recruitment also in “sash” mutants, but the quantitatively dominant contribution is that of MC.

### T-cell recruitment to the infected lungs is diminished in MC-deficient “sash” mutants

**Figure 5:**
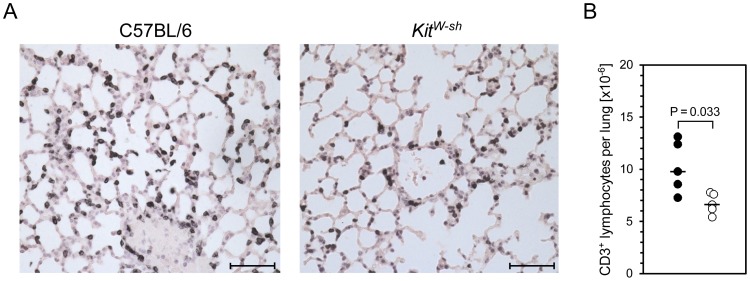
MC-deficiency is associated with less efficient recruitment of T cells to the infected lungs. Pulmonary infiltrates were analyzed 6 days after intravenous infection of MC-sufficient WT C57BL/6 and MC-deficient *Kit^W-sh^* mice. (A) Representative immunohistological images show pulmonary T cell (with a negligible contribution of NKT cells, see Figure 1B) infiltrates *in situ* by black staining of CD3ε, comparing lung tissue sections from WT C57BL/6 (left panel) and *Kit^W-sh^* (right panel) mice. Bar markers represent 50 µm. (B) Comparative analysis of absolute quantities of CD3ε^+^ cells per lung, determined by counting of stained cells in tissue sections extrapolated to the whole organ. Symbols (closed circles: WT C57BL/6; open circles: *Kit^W-sh^* mutant) represent data from individual mice revealing sample sizes and ranges, with the median values indicated.

Having established that MC-deficiency curtails mCMV-induced serum levels of the T and NK/NKT cell-recruiting chemokine CCL5 [27], [29], [30], we quantitated CD3ε^+^ T cells in absolute terms for comparing infiltration of the lungs on day 6 after intravenous infection of WT C57BL/6 mice and MC-deficient “sash” mutants (Fig. 5). As already shown in Fig. 1B, numbers of infiltrating NKT cells, which also express CD3, are negligible in this infection model. At a glance, representative immunohistological low-magnification overview images of lung tissue sections with black staining of CD3ε reveal an apparently lower area density of stained cells in lungs of the MC-deficient “sash” mutant (Fig. 5A). This visual impression is further substantiated and statistically validated by counting labeled cells in tissue sections from lungs of 5 mice per group, extrapolated to absolute numbers of CD3^+^ cells in the whole organ (Fig. 5B). With a median value difference of ca. 3 million CD3^+^ cells per lung, the reduction of lung infiltration by shortness of MC-derived CCL5 is quite substantial in absolute terms. From long experience with CD8 T-cell transfer experiments we know that 10-fold differences in the numbers of transferred cells are required to cause differences in viral replication in lungs of recipient mice, but 10-fold more cells transferred result in only 1.5-fold differences in the numbers of cells that actually infiltrate into lung tissue (J. P., unpublished data), so that the infiltration difference observed here between WT C57BL/6 mice and “sash” mutants is biologically meaningful.

### Impaired virus-triggered CD8 T-cell recruitment in MC-deficient mutant mice can be reversed by reconstitution of MC

Previous work performed in the HCT model of mCMV infection in immunocompromised BALB/c recipients has revealed a fulminant recruitment of newly reconstituted CD8 T cells to the lungs, confining viral spread within lung parenchyma and ultimately resolving viral replication at this pathogenically relevant extralymphoid site [10], [11]. Likewise, despite different host immunogenetics and immune status, in the current model of mCMV infection of immunocompetent C57BL/6 mice, CD8 T cells also were found to predominate in the pulmonary leucocyte population (Fig. 1B). Also shown exemplarily in Fig. 1B are the gating principles for analyzing leucocyte populations, and, in particular, the necessity to exclude CD45^−^ tissue cells from the analysis of lung cell preparations.

**Figure 6:**
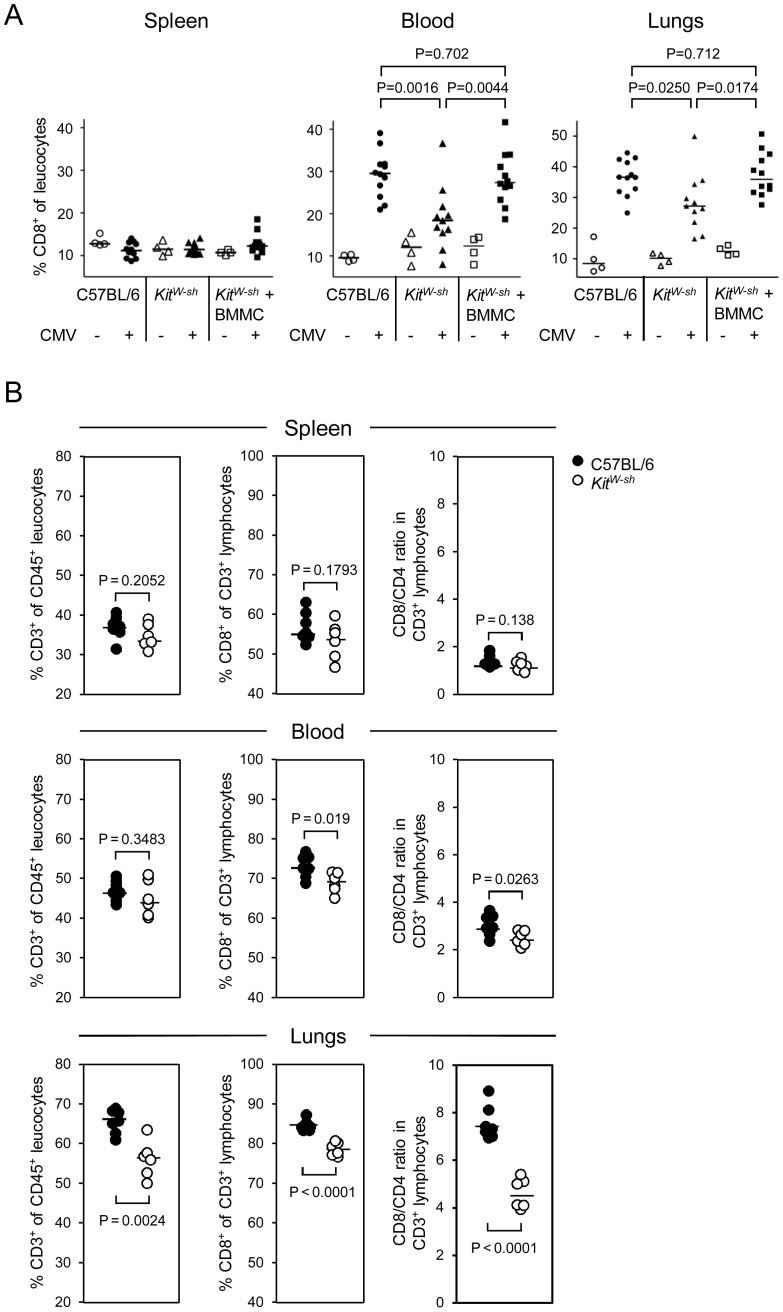
MC deficiency preferentially attenuates the recruitment of CD8 T cells to the lungs and is reversed by MC reconstitution. (A) CD8 T-cell recruitment by infection is curtailed in MC-deficient mice and reversed by MC reconstitution. Shown are results from cytofluorometric analyses (recall Figure 1B for the gating strategy), compiled from two independent experiments, revealing the influence of MC on the proportions of CD8 T cells relative to all leucocytes in lymphoid (spleen) and non-lymphoid (lungs) compartments as well as in the circulation (peripheral blood) on day 6 after intravenous infection. Comparisons were performed in absence or presence of mCMV infection in MC-sufficient WT C57BL/6 mice, MC-deficient *Kit^W-sh^* mice, and (BM)MC-reconstituted *Kit^W-sh^* mice. Symbols represent data from individual mice revealing sample sizes and ranges, with the median values indicated. P values are given for comparisons of interest, unless the difference is obvious. (B) CD8 T-cell recruitment to the lungs suffers preferentially from MC deficiency. Shown are results from an independent experiment focusing on differences between the three compartments regarding the proportions of CD3^+^ T cells among CD45^+^ leucocytes, of CD8^+^ cells among all T cells, and the CD8/CD4 ratios. Note that it is important to calculate CD8/CD4 ratios for each individual separately, as these values are linked in that the individual with the highest percentage of CD8 T cells is necessarily also the one with the lowest percentage of CD4 T cells, and inverse.

To determine the effect of MC-deficiency specifically on the recruitment of CD8 T cells, we quantitated their proportion among all CD45^+^ leucocytes in spleen, blood, and lungs, dependend on infection and presence (C57BL/6 mice) or absence (“sash” mice) of MC (Fig. 6A). Although it appears plausible to propose that the “sash” genotype is responsible for an observed phenotype, firm evidence for a causative role of MC-deficiency demands repair of the deficiency by reconstitution of “sash” mutants with WT MC to exclude a role of any unknown and unintended effect the *Kit^W-sh/W-sh^* mutation might possibly have besides the canonical phenotype of MC-deficiency (Fig. 6A). While the proportion of CD8 T cells in the spleen of WT C57BL/6 mice, “sash” mice, and (BM)MC-reconstituted “sash” mice proved to be generally low and not notably influenced by infection and absence or presence of MC (Fig. 6A, left panel), the export into the circulation (center panel) and the recruitment to the lungs (right panel) were strongly induced by the infection, which is in accordance with the experience made with HCT models of pulmonary mCMV infection [10], [11]. The appearance of elevated numbers of CD8 T cells in the peripheral blood suggests that the elevated levels in pulmonary infiltrates mainly result from recruitment to the lungs rather than from on-site expansion by proliferation within lung interstitium and parenchyma. Levels of CD8 T cells in both peripheral blood and lungs after infection were found to be diminished in MC-deficient “sash” mutants, whereas, importantly, MC reconstitution of “sash” mutants with adoptively transferred BMMC derived from WT C57BL/6 mice restored the CD8 T-cell recruitment to WT levels. It should be noted that CD44^+^CD8^+^ cells showed the same pattern, indicating that antigen-experienced CD44^+^ rather than naïve CD44^−^ CD8 T cells were recruited (data not shown).

Among the CD3^+^ cells, the CD8 T cells preferentially profit from the recruitment-function of MC, as can be seen by comparing the CD8/CD4 T-cell ratios in different compartments after infection of WT C57BL/6 mice and the congenic “sash” mutants (Fig. 6B). While the ratios are balanced in the spleen (ca. 1∶1) and only slightly elevated and marginally segregating between WT and mutant (3∶1 vs. 2.5∶1) in the peripheral blood, ratios are highly elevated and significantly different in the lungs, indicating that CD8 T cells, more than CD4 T cells, are trapped in the lungs.

In summary, this set of experiments provided firm evidence to conclude that the “sash” phenotype of curtailed CD8 T-cell recruitment to the lungs can indeed be attributed to the MC deficiency.

### MC do not affect viral epitope-specificity repertoire, activation state, and effector function of CD8 T cells recruited to the lungs upon infection

The question remained, if, besides the numbers, MC-deficiency in any way affects also the functionality of the recruited CD8 T cells. Provided that the consequence of MC deficiency is only, or at least primarily, a deficient chemokine-mediated recruitment, one would not expect an influence on CD8 T-cell function on a per cell basis. We first compared the hierarchy of viral epitopes recognized by lung-infiltrating CD8 T cells and found no difference between WT C57BL/6 and “sash” mutant mice (Fig. S3A). Accordingly, there also was no notable difference in the proportion of polyclonal lung-derived CD8 T cells, comprising all viral epitope-specificities defined for the H-2^b^ haplotype [52], responding with IFNγ secretion to the recognition of infected cells (Fig. S3B). Note that lower numbers of CD8 T cells recognizing infected cells, as compared to numbers specific for the selected panel of defined epitopes, results from the viral subversion of peptide-MHC-I presentation in infected cells (for more recent reviews, see [53], [54]).

We next compared the activation state of all lung-derived CD8 T cells, as well as that of CD8 T cells specific for two representative epitopes, M45 and M57 (Fig. S4). With only very minor differences, which we do not regard as meaningful, ca. 80% of the CD8 T cells showed the cell surface phenotype CD62L^−^KLRG1^+^ that characterizes short-lived effector cells (SLEC) [55], [56]. Not unexpectedly for day 6 during acute infection, CD62L^+^ central memory cells (TCM) are rare in the total pulmonary CD8 T-cell population and virtually absent in the two viral epitope-specific subsets thereof. Intermediate numbers of CD8 T cells display the phenotype CD62L^−^KLRG1^−^, a population comprised of effector-memory cells (TEM) and early effector cells (EEM) [56].

The high percentages of CD8 T cells with the effector cell phenotype of SLEC predicted these cells are cytolytic, which was confirmed by viral epitope-specific cytolysis of target cells exogenously loaded with saturating concentrations of the corresponding antigenic peptides (Fig. S5). This is in accordance with previous data in the HCT model of mCMV infection in BALB/c mice having shown direct *ex vivo* cytolytic activity of lung-infiltrating CD8 T cells [10] as opposed to CD8 T cells derived from lymphoid tissues. Notably, however, MC-sufficient C57BL/6 mice and MC-deficient “sash” mutants did not differ in this respect.

In conclusion, MC-deficiency does not affect the functionality of lung-infiltrating CD8 T cells.

### MC-deficiency is associated with enhanced virus replication and delayed clearance of productive infection in the lungs that can be reversed by MC reconstitution

**Figure 7:**
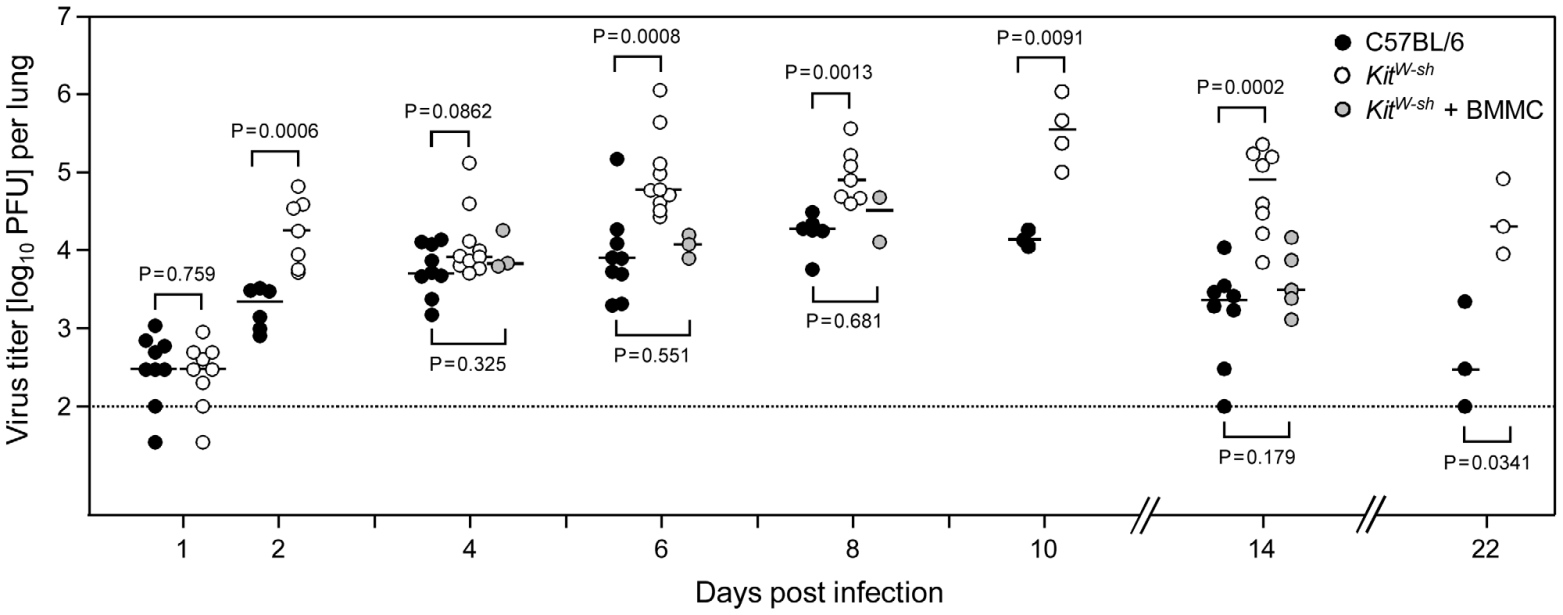
Enhanced virus replication and delayed clearance of productive infection in the lungs associated with MC deficiency are reversed by MC reconstitution. The time course of viral replication in the lungs after intravenous infection is compared between MC-sufficient WT C57BL/6 mice (black-filled circles), MC-deficient *Kit^W-sh^* mice (open circles), and (BM)MC-reconstituted *Kit^W-sh^* mice (gray-filled circles). To increase statistical power and for demonstrating reproducibility, data are compiled from 5 independent experiments. Symbols represent data from individual mice revealing sample sizes and ranges, with the median values indicated. P values are given for comparisons of most interest. Specifically, stable and statistically significant differences between MC-sufficient and MC-deficient mice were established from day 6 onward during the entire remaining observation period, whereas virus replication in MC reconstituted *Kit^W-sh^* mutants was like in WT C57BL/6 mice at all times tested.

We finally investigated the time course of viral replication in the lungs comparing MC-sufficient WT C57BL/6 mice, MC-deficient “sash” mutants, and (BM)MC-reconstituted “sash” mice (Fig. 7). Not unexpectedly, beginning virus replication in the lungs on day 1 is not affected by MC-deficiency, as infection of MC takes a day (recall Fig. 3) and the peak level of CCL5, thought to precede lung infiltration, is reached not until day 2 (recall Fig. 4). Surprisingly, a difference observed on day 2 vanishes by day 4 until it returns. Conspicuously, early virus control coincides with NK activity, before CD8 T cells take over [43]. This might indicate MC-dependence of also of NK activity, a question to be addressed in future work. Viral titers reach a plateau level by day 6 post-infection, the time chosen for most of the preceding analyses, before they begin to decline between days 10 and 14. At all times from day 6 onward, viral replication compared to MC-sufficient WT C57BL/6 mice was significantly enhanced in the MC-deficient, CCL5-deficient, and CD8 T cell-recruitment-deficient “sash” mutants. Importantly, in “sash” mice reconstituted for MC with BMMC, virus replicated in the lungs with no significant difference to the replication in MC-sufficient WT C57BL/6 mice, indicating that enhanced virus replication in “sash” mutants can indeed be attributed to the consequences of MC deficiency.

## Discussion

Antigen-dependent activation of MC by crosslinking of specific membrane-bound IgE antibodies has been so firmly established as the triggering causative event underlying anaphylactic responses that it has long been grossly neglected to even think of MC in a context beyond the pathophysiology of allergy [57].

In searching for physiological MC functions that are rather protective and beneficial for the host, it was noted that the mucosal MC - IgE axis is involved in the expulsion of some parasitic gastrointestinal helminths in rodents, although it can also significantly contribute to intestinal pathology [58]. Since MC are strategically situated at the boundaries between host and environment, it is today more commonly appreciated that MC can serve as important sentinels of the immune system in a broader context [59]. The change of paradigm began with seminal work on acute bacterial infections in which MC were shown to initiate rapid and life-saving inflammatory reactions in murine models [60], [61]. MC can recognize a broad spectrum of exogenous and also endogenous ligands which often trigger danger signals in the host. Examples for the latter include the neuropeptides substance P and neuromedin U, the vasoconstrictory peptide endothelin as well as defensins and IL33 [62], [63].

While a contribution of MC to antibacterial immunity, particularly to gram-negative species, has been well documented using strains of MC-deficient mice [48], *in vivo* evidence for an implication of MC as innate immune sensors in the protective antiviral immune defense has emerged only recently [64], [65], [66] in studies in which a phenotype of MC-deficient mouse mutants could indeed be reversed by MC reconstitution, thereby verifying a causative role of MC.

Specifically, in a mouse model of Dengue virus infection, St. John and colleagues [64] noted an increased viral burden in the draining popliteal lymph node after intraplantar infection of MC-deficient “sash” mice compared to WT mice or “sash” mice reconstituted with MC. The authors linked this MC-dependent containment of the virus to an MC-driven and likely MC-derived chemokine-mediated recruitment of NK1.1^+^CD3^−^ NK cells and more so of NK1.1^+^CD3^+^ NKT cells, a subset of which coexpressed CD8, into the infected skin. The authors' conclusion that NK and NKT cells were consequential to antiviral immunity in this model was supported by the observation of enhanced infection after depletion of NK1.1^+^ cells. However, as MHC class-I-restricted virus-specific CD8 T cells rapidly acquire NK1.1 upon activation [67], [68], a role for canonical CD8 T cells was not rigorously excluded. In a mouse model of vaccinia virus infection of the skin, Wang and colleagues [65] reported an increased susceptibility of MC-deficient “sash” mice that was reversed by MC reconstitution. The authors found MC degranulation is required for the antiviral contribution of MC and concluded that antivirally-active peptides released by MC upon degranulation account for the antiviral effect. Recruitment of antiviral NK, NKT, or T cells to the site of infection was not investigated in that study. In a mouse model of cutaneous herpes simplex virus type-2 (HSV-2) infection, a virus belonging to the alpha-subfamiliy of the herpesviruses, Aoki and colleagues (66) very recently described elevated skin infection and increased overall severity of the infection in MC-deficient mice. The phenotype was reversed by reconstitution with BMMC from WT mice but not with BMMC derived from TNF-ko or IL6-ko mice, from which the authors concluded that these MC-derived cytokines are critically involved in the antiviral contribution made by MC. The virus did not infect BMMC and did not directly induce production of TNFα and IL6 by BMMC *in vitro*; rather, IL33 released by infected keratinocytes induced the production of these cytokines in the MC without inducing degranulation. Notably, MC-deficiency did not curtail the recruitment of inflammatory cells to the cutaneous site of infection and, in the authors' own interpretation, did not notably diminish the CD8 T-cell response to the viral glycoprotein gB.

So, in summary of the literature most pertinent to our work, the three mouse models discussed above share the cutaneous site of infection as well as the finding that virus control is diminished in MC-deficient mice and restored by MC-reconstitution, which reliably identified a causative role of MC in virus containment after local infection. Notably, this was true for members of different virus families, including a positive-strand RNA virus and two double-stranded DNA viruses, which actually have not much in common genetically as well as pathogenetically, so that one must propose a more general innate response of MC to virus infection. Recruitment of effector cells of the innate or adaptive arm of the immune system by MC-derived chemokines, such as CCL5/RANTES, to sites of infection could be such a common mechanism for the contribution of MC to virus containment. It is a shortcoming of previous mouse models of MC involvement in antiviral control that virus infection was not studied in the natural host, which bears a risk of investigating virus-host interactions that are not balanced by evolutionary co-speciation and mutual adaptation. This gives a distinctive advantage to the model of the highly species-specific murine CMV, a member of the beta-subfamily of the herpesviruses, in its natural host [69]. In addition, studying the role of MC in controlling pulmonary infection addresses the most relevant clinical manifestation of CMV disease, namely interstitial pneumonia, so that the results can make a prediction for translational validation in clinical settings.

In accordance with the three models discussed above, mCMV infection of the natural host also revealed an MC-dependent control of infection that, accordingly, was curtailed in MC-deficient mice and restored by MC reconstitution. Thus, there is no doubt of a causative role of MC instead of other c-Kit related mechanisms of immunomodulation [70]. The new finding here is that such a role for MC is not restricted to cutaneous sites rich in skin-resident MC but applies also to the lungs in the course of a systemic infection. As we have shown here, virus replication at an advanced stage of pulmonary infection of C57BL/6 mice is controlled by antiviral effector CD8 T cells forming protective nodular inflammatori foci to sequester infected lung cells and prevent virus spread within alveolar epithelium, whereas a contribution of Ly49H^+^ NK cells activated by the virus-encoded ligand m157 is limited to earlier stages. In this context, it is important that MC-deficiency was found here to be associated with reduced virus-induced serum levels of chemokine CCL5 and diminished recruitment of T lymphocytes, in particular of CD8 T cells, to the infected lungs with consequent enhancement of viral replication. MC-deficiency, however, though it significantly reduced the quantity of lung-infiltrating CD8 T cells to a histologically obvious degree, did not abolish virus-induced CD8 T-cell infiltration and antiviral control. The explanation for this may come from our observation of moderately but more stably elevated serum levels of the CD8 T cell-recruiting chemokine CCL5 also in infected MC-deficient “sash” mutants when compared to the pre-infection levels that were found to be identical for WT C57BL/6 mice and the congenic “sash” mutants. This suggests the existence of additional other cellular sources of CCL5. Yet, a strong early wave of CCL5 proved to be MC-derived and is likely important for the kick-start of an efficient CD8 T-cell infiltration and consequently reduced peak viral load and accelerated clearance of the productive infection of the lungs. Notably, even in MC-sufficient WT C57BL/6 mice, NK cells were virtually absent in infected lungs and NKT cells were not prominent either. In this respect, the mCMV model appears to differ markedly from the model of cutaneous Dengue virus infection [64], where MC-mediated recruitment of NK cells and NKT cells correlated with antiviral control.

The question remained how virus, directly or indirectly, activates MC, a problem where we learned from errors. Reminiscent to the observation by Aoki and colleagues [66] that HSV-2 does not infect cell culture-propagated BMMC *in vitro* and also fails to induce degranulation or activate cytokine production, we could not detect expression of viral proteins or fluorescent GFP in BMMC incubated with mCMV or mCMV-GFP reporter virus, respectively, and BMMC also were not activated for degranulation (negative data not shown). We thus concluded, falsely as we know now, that MC are resistant to mCMV infection like they are seemingly resistant to HSV-2. Important in this context are findings by Matsushima and colleagues [32] who showed TLR-mediated production of cytokines and chemokines from *ex vivo* skin-derived MC but not from BMMC, indicating that BMMC may not be an always reliable source for drawing general conclusions on MC functions. In fact, as we show here with reporter virus mCMV-GFP, intraperitoneal infection of mice infects FcεRI^+^CD117^+^ MC *in vivo*. Most importantly, MC degranulation, as indicated by expression of CD107a, was strictly limited to GFP-expressing, infected MC. These data identified MC as a first-hit target cell-type for mCMV infection *in vivo* and also revealed that infection of MC by mCMV is required for triggering MC degranulation *in vivo*. If direct infection is also sufficient for MC degranulation or requires MC-conditioning cytokine(s) from third-party cells as co-factor(s), and which signaling pathways in MC are activated by mCMV, are exciting questions that await further investigation.

In conclusion, these data have revealed an hitherto unconsidered role of MC in recruiting antiviral CD8 T cells more efficiently to a relevant organ site of CMV disease, and thus predict that MC supplementation in recipients of hematopoietic (stem) cell transplantation could improve control of a primary or reactivated CMV infection for prevention of CMV pneumonia and, presumably, also of other organ manifestations of CMV disease.

## Materials and Methods

### Mice

Genetically MC-deficient *Kit^W-sh/W-sh^* (briefly *Kit^W-sh^*) “sash” mice [35], [36], [37] and C57BL/6 mice as congenic WT controls were bred and housed under specified pathogen-free conditions at the Central Laboratory Animal Facility (CLAF) at the University Medical Center of the Johannes Gutenberg-University, Mainz, Germany.

### Ethics statement

Animal research protocols were approved by the ethics committee of the Landesuntersuchungsamt Rheinland-Pfalz, permission no. 23 177-07/G09-1-004, according to German Federal Law §8 Abs. 1 TierSchG (animal protection law).

### Peptides

Custom peptide synthesis to a purity of >80% was performed by JPT Peptide Technologies (Berlin, Germany). Synthetic mCMV-peptides M38 (SSPPMFRVP), M45 (HGIRNASFI), M57 (SCLEFWQRV), m139 (TVYGFCLL), and m141 (VIDAFSRL) were used for exogenous loading of target cells in the ELISpot assay and in the cytolytic assay.

### Procedures of infection

(i) Infection of mice. Intravenous infection of 8- to 20-week-old *Kit^W-sh^* or C57BL/6 mice (both haplotype H-2^b^) was performed at the tail vein with 10^6^ PFU/500 µl PBS per mouse of cell culture-purified, bacterial artificial chromosome (BAC)-derived mCMV MW97.01 [71], here briefly referred to as mCMV, or mutant virus mCMV-Δm157 lacking the ligand of the activatory NK cell receptor Ly49H [45]. For the measurement of MC degranulation *in vivo* (see below), mice were infected intraperitoneally with 10^6^ PFU/100 µl PBS of reporter virus mCMV-GFP [38]. (ii) Infection of cells. C57BL/6-derived mouse embryo fibroblasts (MEF) were prepared as described previously, and were centrifugally infected with 0.2 PFU per cell, which results in an MOI of 4 [72]. Before use as stimulator cells in the ELISpot assay (see below), infected MEF were incubated for another 60 min.

### Quantitation of chemokine CCL5/RANTES

To measure serum levels of CCL5/RANTES, mice were infected intravenously with 10^6^ PFU of mCMV. Blood was collected from the tail vein pre-infection (d0) or on days 1, 2, 3, and 4 post-infection in BD Microtainer tubes (catalog no. 965951, BD Biosciences, Heidelberg, Germany). Sera were purified according to the supplier's instruction. CCL5/RANTES levels for individual mice were determined in duplicates of 50 µl of non-diluted sera with the Mouse RANTES Instant ELISA (catalog no. BMS6009INST, eBioscience, Frankfurt, Germany), according to the manufacturer's instructions.

### Quantitation of infection in host tissues and CD8 T-cell depletion

Virus replication in lungs, spleen, and salivary glands was assessed by quantification of infectious virus as plaque-forming units (PFU) present in the respective organ homogenates by using virus plaque assay on subconfluent second-passage MEF monolayers with the technique of centrifugal enhancement of infectivity, as described in greater detail elsewhere [72]. For determining the role of CD8 T cells in the control of infection, virus titers were determined in organs of mice depleted of CD8 T cells on day -1 prior to infection by intravenous administration of monoclonal antibody YTS169.4 as described previously [15], [73], [74].

### Generation of BMMC and reconstitution of MC-deficient mice

Bone marrow (BM)-derived mast cells (BMMC) from WT C57BL/6 mice were generated by using standard procedures [75]. In brief, BM cells were cultivated in IMDM, supplemented with 10% FCS (inactivated at 56°C), 2 mM L-glutamine, 1 mM pyruvate, 5×10^−5^M 2-ME, 100 U/ml penicillin, 100 µg/ml streptomycin, 20 U/ml mIL-3, 200 ng/ml SCF and 50 U/ml mIL-4. Nonadherent cells were transferred to fresh culture plates every 2–3 days to remove adherent macrophages and fibroblasts. After 4 weeks of culture, the resulting BMMC population had reached a purity of more than 99% and was used for MC reconstitution of MC-deficient *Kit^W-sh^* mice. To this end, BMMC were washed twice in PBS, and 5×10^6^ MC/200 µl PBS per mouse were injected intravenously. Experiments were conducted 16 weeks later. We and others have repeatedly shown that this protocol leads to the development of normal MC numbers in most organs, specifically also in the lungs. [36], [37], [76], [77].

### Preparation of leucocytes

Pulmonary mononuclear cells were isolated essentially as described previously ([10], [72] and references therein) with minor modification. In brief, mice were lethally anesthetized by carbon dioxide inhalation. The lungs were perfused via the right ventricle and excised. Tracheae, bronchi, and pulmonary lymph nodes were discarded and the lung lobes were minced. Digestion of tissue from 3–5 lungs was performed in 15 ml of supplemented DMEM, containing collagenase A (1.6 mg/ml; catalog no. 10 103 586 001, Roche, Mannheim, Germany) and DNase I (50 µg/ml; catalog no. DN-25, Sigma-Aldrich, Steinheim, Germany), for 1 h at 37°C with constant stirring. Mononuclear cells were enriched by density gradient centrifugation for 30 min at 760×*g* on Lymphocyte Separation Medium 1077 (catalog no. J15-004, PAA, Cölbe, Germany). Leucocytes from the spleen were prepared according to standard protocols. Blood leucocytes were isolated by mixing the blood samples with PBS containing 30 mM EDTA to prevent coagulation. Erythrocytes were lysed with hypotonic ACK buffer (catalog no. A10492-01, life technologies, Darmstadt, Germany) according to manufacturer's instructions. Bronchoalveolar lavage (BAL) was performed and BAL leucocytes were isolated as described in detail by Maxeiner and colleagues [78]. Erythrocytes in the BAL cell pellet were lysed by using Red Blood Cell Lysing Buffer (catalog no. R7757, Sigma-Aldrich) according to the manufacturer's instructions.

### Cell surface phenotype analyses of leucocyte populations

Cytofluorometric analyses were performed with flow cytometer FC500 and CXP analysis software V2.2 (Beckman Coulter, Krefeld, Germany). Single cell suspensions were prepared from organs or peripheral blood (see above), unspecific staining was blocked with unconjugated anti-FcγRII/III, and cells were stained in multi-color combinations with the following antibodies for cytofluorometric analyses of leucocyte subpopulations: FITC-conjugated anti-CD3ε (clone 145-2C11; Beckman Coulter; FL-1), PE-conjugated anti-CD4 (clone GK1.5; eBioscience; FL-2), PE-CF594-conjugated anti-CD8a (clone 53–6.7; BD Biosciences; FL-3), APC-conjugated anti CD19 (clone 1D3; eBioscience; FL-4), PE-Cy7-conjugated anti-CD45 (clone 30-F11; eBioscience; FL-5). and PE-conjugated anti-CD335 (alternative designation: NKp46, clone 29A1.4; eBioscience; FL-2).

For quantitating M57 epitope-specific CD8 T cells in BAL (see above), unspecific staining was blocked as described above and specific staining was performed with PE-Cy7-conjugated anti-CD45 (see above; FL-5), FITC-conjugated anti-CD8a (clone 53–6.7; BD Biosciences; FL-1), and PE-conjugated MHC-peptide Dextramer H-2Kb-M57 (SCLEFWQRV) [10 µl per 10^6^ cells; code no. JD3446-PE, Immudex, Copenhagen, Denmark; FL-2) for detecting cells carrying an M57-specific T cell receptor (M57-TCR).

For determining the activation state of all as well as of viral epitope-specific CD8 T cells, staining was performed with FITC-conjugated anti-CD8a (clone 53–6.7; BD Biosciences; FL-1), PE-conjugated anti-KLRG1 (clone 2F1; eBioscience; FL-2), PE-Cy7-conjugated anti-CD62L (clone MEL-14; Beckman Coulter; FL-5) and APC-conjugated MHC-peptide Dextramer H-2Db-M45 (HGIRNASFI) [10 µl per 10^6^ cells; code no. JA3445-APC, Immudex, FL-4] or APC-conjugated MHC-peptide Dextramer H-2Kb-M57 (SCLEFWQRV) [10 µl per 10^6^ cells; code no. JD3446-APC, Immudex; FL-4]. Control staining for an unrelated ovalbumin peptide-specific TCR (Control-TCR) was performed with APC-conjugated MHC-peptide Dextramer H-2Kb-SIINFEKL (10 µl per 10^6^ cells; code no. JD2163-APC, Immudex; FL-4).

### Measurement of MC activation *in vivo*

At 4 or 24 hours after intraperitoneal infection with mCMV-GFP [38], mice were sacrificed by cervical dislocation, and peritoneal lavage was performed with 10 ml of ice-cold PBS. Unspecific staining was blocked with unconjugated anti-FcγRII/III, and PEC were stained with the following antibodies for cytofluorometric analysis: AlexaFluor780-conjugated anti-CD117 (clone ACK2, eBioscience) and PE-Cy7-conjugated anti-FcεRI (clone MAR-1; BioLegend, San Diego, CA, USA) and, in assays of degranulation, also with AlexaFluor647-conjugated anti-CD107a (clone 1D4B; eBioscience). Analyses were performed with an LSRII flow cytometer (BD Biosciences) and BD FACSDiva software V6.1.3.

### ELISpot assays

Frequencies of virus-specific CD8 T cells were determined by IFNγ-based ELISpot assays as described ([79], [80], and references therein). In brief, graded numbers of effector cells were sensitized for IFNγ secretion in triplicate assay cultures with antigen presenting stimulator cells. For quantitating virus epitope-specific effector cells, CD8 T cells were incubated with EL-4 (H-2^b^) lymphoma cells as stimulator cells exogenously loaded with synthetic antigenic peptides at saturating concentrations. Numbers of virus-specific CD8 T cells capable of recognizing infected cells were quantitated using mCMV-infected MEF (see above) as stimulator cells presenting naturally-processed antigenic viral peptides. Frequencies of IFNγ-secreting, spot-forming cells and the corresponding 95% confidence intervals were calculated by intercept-free linear regression analysis. Spots were counted automatically based on standardized criteria using Immunospot S4 Pro Analyzer (CTL, Shaker Hights, OH, USA) and CTL-Immunospot software V5.1.36.

### Cytolysis assay

Cytolytic activity of immunomagnetically-purified pulmonary CD8 T cells was tested in a standard 4 h [^51^Cr]-release assay performed with 1000 [^51^Cr]-labeled target cells per assay culture at the E∶T ratios indicated. Target cells were EL-4 (H-2^b^) lymphoma cells exogenously loaded with synthetic mCMV peptides at optimized concentrations. For each E∶T ratio, the “low controls” of [^51^Cr]-release were determined with EL-4 cells not loaded with antigenic peptide. Data thus represent the percentage of epitope-specific lysis and are given as the mean value for triplicate cultures.

### Immunohistological analyses

Pulmonary T cell-infiltrates *in situ* were visualized by black staining of cells expressing CD3ε in 2-µm lung tissue sections using the peroxidase-diaminobenzidine (DAB)-nickel method described in great detail previously [81]. For determining absolute numbers of CD3ε^+^ cells per lung, numbers counted for representative 10-mm^2^ areas of sections were extrapolated to the whole lung using the multiplication factor *V/v*×*d/D* with *V* = volume of a paraffin-embedded lung (0.135 cm^3^), *v* = volume of counted section area (10 mm^2^×0.002 mm), *d* = thickness of section (0.002 mm), and *D* = diameter of a T cell (0.01 mm) [11].

Two-color contextual immunohistological detection of CD3ε^+^ T cells (red staining) and infected cells expressing the intranuclear viral protein IE1 (black staining) was performed essentially as described by us in a methods book contribution [81], except that colors are inverted by using ABC-peroxidase DAB-nickel staining for IE1 and an indirect staining of CD3ε, using alkaline phosphatase (AP)-conjugated anti-rat IgG and Fuchsin as substrate.

Two-color immunohistological localization of CD31/PECAM-1^+^ endothelial cells and CD3^+^ T cells was started with a rat monoclonal antibody directed against murine CD31 (clone SZ31; catalog no. DIA-310), DIANOVA, Hamburg, Germany) as the first antibody, followed by biotin-conjugated polyclonal anti-rat Ig antibody (catalog. no. 554014, BD Biosciences) and the peroxidase-coupled avidin biotin complex (Vectastain Elite ABC Kit, catalog. no. PK-6100, Vector Laboratories, Peterborough, UK). As substrate, 3,3′-diaminobenzidine tetrahydrochloride (catalog no. D-5637, Sigma-Aldrich) was used, followed by color-enhancement with ammonium-nickelsulphate-hexahydrate (catalog. no. 09885, Sigma-Aldrich) for black staining of the endothelial cells. In a second round, CD3^+^ T cells were labeled with a rat monoclonal antibody directed against CD3ε (clone CD3-12; catalog. no. SM1754P, acris antibodies, Hiddenhausen, Germany), followed by the biotin-conjugated antibody and the peroxidase-coupled avidin biotin complex as above. To prevent cross-binding of the secondary antibody in the second round to cells stained already in the first round, digestion with trypsin was included in between both rounds. Finally, turquoise-green staining of CD3^+^ cells was achieved by using the HRP-Green Solution Set (catalog no. S-99055-103, 42 life sciences, Bremerhafen, Germany). Throughout, light counterstaining was performed with hematoxylin.

### Statistical analysis

In assays for which data are known to be normally (cell numbers and percentages, chemokine concentrations) or log-normally (virus titers) distributed, P values were calculated for the linear or the log-transformed data sets, respectively, by unpaired two-tailed Student's t test with Welch's correction not assuming equal variance. Differences are considered significant if P<0.05 and highly significant if P<0.01.

## Supporting Information

Figure S1 **Ly49H^+^ NK cells stimulated by viral ligand m157 are not involved in the control of pulmonary infection at a later stage.** C57BL/6 mice were infected either with WT virus or with mutant virus mCMV-Δm157 lacking the viral gene m157 that encodes a potent ligand of the activatory NK cell receptor Ly49H. Virus titers in the indicated organs were determined on day 14 post-infection. Dots represent virus titers in individual mice revealing the sample sizes and ranges, with the median values indicated. The dotted lines indicate the detection limits.
(TIF)

Figure S2 **Cytofluorometric quantitation of viral epitope-specific CD8 T cells recovered by BAL.** Corresponding to the data shown in Figure 2, leucocytes retrieved by BAL were analyzed cytofluorometrically. A gate was set on CD45^+^CD8^+^ T cells, and within this population, cells carrying a TCR specific for the M57 epitope (M57-TCR) were identified by binding of fluorochrome-conjugated peptide-MHC class-I (SCLEFWQRV-H-2K^b^) multimer. Shown are color-coded density plots with red and blue representing highest and lowest density, respectively. (FL) fluorescence channel and fluorescence intensity. Percentages of main interest are indicated for gated areas.
(TIF)

Figure S3 **MC deficiency has no influence on the viral epitope hierarchy of the CD8 T-cell response in the lungs nor on recognition of infected cells.** MC-sufficient WT C57BL/6 (black bars) and MC-deficient *Kit^W-sh^* (open bars) mice were infected intravenously. On day 6 post-infection, immunomagnetically-purified CD8 T lymphocytes derived from the lungs of 6 mice per group were used as responder cells in IFNγ-based ELISpot assays. (A) Hierarchy of viral epitopes recognized by pulmonary CD8 T cells. EL-4 stimulator cells were exogenously loaded with synthetic peptides representing the indicated MHC-I-presented epitopes. (Ø) stimulator cells with no peptide added. (B) Recognition of infected cells (MEF) presenting naturally-processed viral peptides despite the expression of viral immune evasion genes. (n.i.) uninfected MEF. Throughout, frequencies of responding, IFNγ-secreting cells were determined by intercept-free linear regression analysis. Bars represent most probable numbers, and error bars indicate 95% confidence intervals.
(TIF)

Figure S4 **MC deficiency does not notably alter the activation phenotype of pulmonary CD8 T cells.** MC-sufficient WT C57BL/6 (left panels) and MC-deficient *Kit^W-sh^* (right panels) mice were infected intravenously. Multi-color cytofluorometric analyses were performed on day 6 post-infection for lung infiltrate cells pooled from 6 mice per group and pre-gated on CD8^+^ cells (for the gating strategy, recall Fig. 1B). Shown are color-coded density plots with red and blue representing highest and lowest density, respectively. The analyzed cell surface markers are indicated. Viral epitope-specific CD8 T cells were identified with TCR-specific peptide-MHC class-I multimers, specifically with M45-D^b^ and M57-K^b^ Dextramers. (FL) fluorescence channel and fluorescence intensity. Percentages of main interest are indicated for gated areas and quadrants.
(TIF)

Figure S5 **MC deficiency does not alter the** ***ex vivo*** **cytolytic activity of pulmonary CD8 T cells.** Immunomagnetically-purified CD8 T cells from infected MC-sufficient WT C57BL/6 and MC-deficient *Kit^W-sh^* mice were derived from the experiment described in Figure S3, so that the different effector functions can be directly compared. Here, the CD8 T cells were analyzed *ex vivo* for their cytolytic effector function with no preceding expansion in cell culture. Viral epitope-specific cytolysis was assayed at graded effector-to-target (E∶T) cell ratios with EL-4 lymphoma cells as target cells pulsed with saturating concentrations of synthetic peptides corresponding to the viral epitopes indicated.
(TIF)

## References

[1] Hebart H, Einsele H (2004) Clinical aspects of CMV infection after stem cell transplantation. Human Immunol 65: 432–436. 15172442 10.1016/j.humimm.2004.02.022

[2] Seo S, Boeckh M (2013) Clinical cytomegalovirus research: haematopoietic cell transplantation. In: Reddehase MJ, editor. Cytomegaloviruses: from molecular pathogenesis to intervention. Caister Academic Press, Norfolk, UK. Vol. II: pp337–353.

[3] Mayer A, Podlech J, Kurz S, Steffens HP, Maiberger S, et al (1997) Bone marrow failure by cytomegalovirus is associated with an in vivo deficiency in the expression of essential stromal hemopoietin genes. J Virol 71: 4589–4598. 9151853 10.1128/jvi.71.6.4589-4598.1997 PMC191681

[4] Steffens HP, Podlech J, Kurz S, Angele P, Dreis D, et al (1998) Cytomegalovirus inhibits the engraftment of donor bone marrow cells by downregulation of hemopoietin gene expression in recipient stroma. J Virol 72: 5006–5015. 9573270 10.1128/jvi.72.6.5006-5015.1998 PMC110063

[5] Holtappels R, Ebert S, Podlech J, Fink A, Böhm V, et al. (2013) Murine model for cytoimmuntherapy of CMV disease after haematopoietic cell transplantation. In: Reddehase MJ, editor. Cytomegaloviruses: from molecular pathogenesis to intervention. Caister Academic Press, Norfolk, UK. Vol. II: pp354–381.

[6] Quabeck K (1994) The lung as a critical organ in marrow transplantation. Bone Marrow Transplant 14: 19–28. 7728120

[7] Ljungman P (1995) Cytomegalovirus pneumonia: presentation, diagnosis, and treatment. Semin Respir Infect 10: 209–215. 8668848

[8] Riddell SR (1995) Pathogenesis of cytomegalovirus pneumonia in immunocompromised hosts. Semin Respir Infect 10: 199–208. 8668847

[9] Reddehase MJ, Weiland F, Münch K, Jonjic S, Lüske A, et al (1985) Interstitial murine cytomegalovirus pneumonia after irradiation: characterization of cells that limit viral replication during established infection of the lungs. J Virol 55: 264–273. 2991554 10.1128/jvi.55.2.264-273.1985 PMC254929

[10] Holtappels R, Podlech J, Geginat G, Steffens HP, Thomas D, et al (1998) Control of murine cytomegalovirus in the lungs: relative but not absolute immunodominance of the immediate-early 1 nonapeptide during the antiviral cytolytic T-lymphocyte response in pulmonary infiltrates. J Virol 72: 7201–7212. 9696814 10.1128/jvi.72.9.7201-7212.1998 PMC109942

[11] Podlech J, Holtappels R, Pahl-Seibert MF, Steffens HP, Reddehase MJ (2000) Murine model of interstitial cytomegalovirus pneumonia in syngeneic bone marrow transplantation: persistence of protective pulmonary CD8-T-cell infiltrates after clearance of acute infection. J Virol 74: 7496–7507. 10906203 10.1128/jvi.74.16.7496-7507.2000 PMC112270

[12] Balthesen M, Messerle M, Reddehase MJ (1993) Lungs are a major organ site of cytomegalovirus latency and recurrence. J Virol 67: 5360–5366. 8394453 10.1128/jvi.67.9.5360-5366.1993 PMC237936

[13] Kurz S, Steffens HP, Mayer A, Harris JR, Reddehase MJ (1997) Latency versus persistence or intermittent recurrences: evidence for a latent state of murine cytomegalovirus in the lungs. J Virol 71: 2980–2987. 9060657 10.1128/jvi.71.4.2980-2987.1997 PMC191426

[14] Kurz SK, Reddehase MJ (1999) Patchwork pattern of transcriptional reactivation in the lungs indicates sequential checkpoints in the transition from murine cytomegalovirus latency to recurrence. J Virol 73: 8612–8622. 10482614 10.1128/jvi.73.10.8612-8622.1999 PMC112881

[15] Podlech J, Holtappels R, Wirtz N, Steffens HP, Reddehase MJ (1998) Reconstitution of CD8 T cells is essential for the prevention of multiple-organ cytomegalovirus histopathology after bone marrow transplantation. J Gen Virol 79: 2099–2104. 9747717 10.1099/0022-1317-79-9-2099

[16] Seckert CK, Grießl M, Büttner JK, Freitag K, Lemmermann NA, et al. (2013) Immune surveillance of cytomegalovirus latency and reactivation in murine models: link to ‘Memory Inflation’. In: Reddehase MJ, editor. Cytomegaloviruses: from molecular pathogenesis to intervention. Caister Academic Press, Norfolk, UK. Vol. I: pp374–416.

[17] Alterio de Goss M, Holtappels R, Steffens HP, Podlech J, Angele P, et al (1998) Control of cytomegalovirus in bone marrow transplantation chimeras lacking the prevailing antigen-presenting molecule in recipient tissues rests primarily on recipient-derived CD8 T cells. J Virol 72: 7733–7744. 9733809 10.1128/jvi.72.10.7733-7744.1998 PMC110079

[18] Sacher T, Podlech J, Mohr CA, Jordan S, Ruzsics Z, et al (2008) The major virus-producing cell type during murine cytomegalovirus infection, the hepatocyte, is not the source of virus dissemination in the host. Cell Host Microbe 3: 263–272. 18407069 10.1016/j.chom.2008.02.014

[19] Stahl FR, Heller K, Halle S, Keyser KA, Busche A, et al (2013) Nodular inflammatory foci are sites of T cell priming and control of murine cytomegalovirus infection in the neonatal lung. PLoS Pathog 9: e1003828. 24348257 10.1371/journal.ppat.1003828 PMC3861546

[20] Böhm V, Podlech J, Thomas D, Deegen P, Pahl-Seibert MF, et al (2008) Epitope-specific in vivo protection against cytomegalovirus disease by CD8 T cells in the murine model of preemptive immunotherapy. Med Microbiol Immunol 197: 135–144. 18340461 10.1007/s00430-008-0092-3

[21] Reddehase MJ, Rothbard JB, Koszinowski UH (1989) A pentapeptide as minimal antigenic determinant for MHC class I-restricted T lymphocytes. Nature 337: 651–653. 2465495 10.1038/337651a0

[22] Simon CO, Holtappels R, Tervo HM, Böhm V, Däubner T, et al (2006) CD8 T cells control cytomegalovirus latency by epitope-specific sensing of transcriptional reactivation. J Virol 80: 10436–10456. 16928768 10.1128/JVI.01248-06 PMC1641801

[23] Reddehase MJ, Koszinowski UH (1991) Redistribution of critical major histocompatibility complex and T cell receptor-binding functions of residues in an antigenic sequence after biterminal substitution. Eur J Immunol 21: 1697–1701. 2060579 10.1002/eji.1830210717

[24] Lemmermann NA, Gergely K, Böhm V, Deegen P, Däubner T, et al (2010) Immune evasion proteins of murine cytomegalovirus preferentially affect cell surface display of recently generated peptide presentation complexes. J Virol 84: 1221–1236. 19906905 10.1128/JVI.02087-09 PMC2812335

[25] Lemmermann NA, Kropp KA, Seckert CK, Grzimek NK, Reddehase MJ (2011) Reverse genetics modification of cytomegalovirus antigenicity and immunogenicity by CD8 T-cell epitope deletion and insertion. J Biomed Biotechnol 2011: 812742. 21253509 10.1155/2011/812742 PMC3021883

[26] Sacher T, Andrassy J, Kalnins A, Dölken L, Jordan S, et al (2011) Shedding light on the elusive role of endothelia cells in cytomegalovirus dissemination. PLoS Pathog 7: e1002366. 22114552 10.1371/journal.ppat.1002366 PMC3219709

[27] Galkina E, Thatte J, Dabak V, Williams MB, Ley K, et al (2005) Preferential migration of effector CD8+ T cells into the interstitium of the normal lung. J Clin Invest 115: 3473–3483. 16308575 10.1172/JCI24482 PMC1288831

[28] Zlotnik A, Yoshie O (2000) Chemokines: a new classification system and their role in immunity. Immunity 12: 121–127. 10714678 10.1016/s1074-7613(00)80165-x

[29] McAlpine SM, Issekutz TB, Marshall JS (2012) Virus stimulation of human mast cells results in the recruitment of CD56+ T cells by a mechanism dependent on CCR5 ligands. FASEB J 26: 1280–1289. 22125314 10.1096/fj.11-188979

[30] Orinska Z, Bulanova E, Budagian V, Metz M, Maurer M, et al (2005) TLR3-induced activation of mast cells modulates CD8+ T-cell recruitment. Blood 106: 978–987. 15840693 10.1182/blood-2004-07-2656

[31] Rodewald HR, Feyerabend TB (2012) Widespread immunological functions of mast cells: fact or fiction? Immunity 37: 13–24. 22840840 10.1016/j.immuni.2012.07.007

[32] Matsushima H, Yamada N, Matsue H, Shimada S (2004) TLR3-, TLR7-, and TLR9-mediated production of proinflammatory cytokines and chemokines from murine connective tissue type skin-derived mast cells but not from bone marrow-derived mast cells. J Immunol 173: 531–541. 15210814 10.4049/jimmunol.173.1.531

[33] Kulka M, Metcalfe DD (2006) TLR3 activation inhibits human mast cell attachment to fibronectin and vitronectin. Mol Immunol 43: 1579–1586. 16280166 10.1016/j.molimm.2005.09.019

[34] Sandig H, Bulfone-Paus S (2012) TLR signaling in mast cells: common and unique features. Front Immunol 3: 185 doi:10.3389/fimmu.2012.00185 22783258 10.3389/fimmu.2012.00185 PMC3389341

[35] Lyon MF, Glenister PH (1982) A new allele sash (Wsh) at the W-locus and a spontaneous recessive lethal in mice. Genet Res 39: 315–322. 7117838 10.1017/s001667230002098x

[36] Grimbaldeston MA, Chen CC, Piliponsky AM, Tsai M, Tam SY, et al (2005) Mast cell-deficient W-sash c-kit mutant Kit W-sh/W-sh mice as a model for investigating mast cell biology in vivo. Am J Pathol 167: 835–848. 16127161 10.1016/S0002-9440(10)62055-X PMC1698741

[37] Wolters PJ, Mallen-St Clair J, Lewis CC, Villalta SA, Baluk P, et al (2005) Tissue-selective mast cell reconstitution and differential lung gene expression in mast cell-deficient Kit(W-sh)/Kit(W-sh) sash mice. Clin Exp Allergy 35: 82–88. 15649271 10.1111/j.1365-2222.2005.02136.x PMC2271075

[38] Angulo A, Ghazal P, Messerle M (2000) The major immediate-early gene ie3 of mouse cytomegalovirus is essential for viral growth. J Virol 74: 11129–11136. 11070009 10.1128/jvi.74.23.11129-11136.2000 PMC113196

[39] Jonjic S, Mutter W, Weiland F, Reddehase MJ, Koszinowski UH (1989) Site-restricted persistent cytomegalovirus infection after selective long-term depletion of CD4+ T lymphocytes. J Exp Med 169: 1199–1212. 2564415 10.1084/jem.169.4.1199 PMC2189231

[40] Campbell AE, Cavanaugh VJ, Slater JS (2008) The salivary glands as a privileged site of cytomegalovirus immune evasion and persistence. Med Microbiol Immunol 197: 205–213. 18259775 10.1007/s00430-008-0077-2

[41] Arase H, Mocarski ES, Campbell AE, Hill AB, Lanier LL (2002) Direct recognition of cytomegalovirus by activating and inhibitory NK cell receptors. Science 296: 1323–1326. 11950999 10.1126/science.1070884

[42] Smith HRC, Heusel JW, Mehta IK, Kim S, Dorner BG, et al (2002) Recognition of a virus-encoded ligand by a natural killer cell activation receptor. Proc Natl Acad Sci USA 99: 8826–8831. 12060703 10.1073/pnas.092258599 PMC124383

[43] Mitrovic M, Arapovic J, Jordan S, Fodil-Cornu N, Ebert S, et al (2012) The NK cell response to mouse cytomegalovirus infection affects the level and kinetics of the early CD8(+) T-cell response. J Virol 86: 2165–2175. 22156533 10.1128/JVI.06042-11 PMC3302391

[44] Walzer T, Bléry M, Chaix J, Fuseri N, Chasson L, et al (2007) Identification, activation, and selective in vivo ablation of mouse NK cells via NKp46. Proc Natl Acad Sci USA 104: 3384–3389. 17360655 10.1073/pnas.0609692104 PMC1805551

[45] Bubic I, Wagner M, Krmpotic A, Saulig T, Kim S, et al (2004) Gain of virulence caused by loss of a gene in murine cytomegalovirus. J Virol 78: 7536–7544. 15220428 10.1128/JVI.78.14.7536-7544.2004 PMC434107

[46] Anderson KG, Sung H, Skon CN, Lefrancois L, Deisinger A, et al (2012) Cutting edge: intravascular staining redefines lung CD8 T cell responses. J Immunol 189: 2702–2706. 22896631 10.4049/jimmunol.1201682 PMC3436991

[47] Jarvis MA, Nelson JA (2007) Human cytomegalovirus tropism for endothelial cells: not all endothelial cells are created equal. J Virol 81: 2095–2101. 16956936 10.1128/JVI.01422-06 PMC1865914

[48] Abraham SN, St John AL (2010) Mast cell-orchestrated immunity to pathogens. Nat Rev Immunol 10: 440–452. 20498670 10.1038/nri2782 PMC4469150

[49] Munks MW, Cho KS, Pinto AK, Sierro S, Klenerman P, et al (2006) Four distinct patterns of memory CD8 T cell responses to chronic murine cytomegalovirus infection. J Immunol 177: 450–458. 16785542 10.4049/jimmunol.177.1.450

[50] Gibbons AE, Price P, Robertson TA, Papadimitriou JM, Shellam GR (1990) Replication of murine cytomegalovirus in mast cells. Arch Virol 115: 299–307. 2175592 10.1007/BF01310538

[51] Wagner FM, Brizic I, Prager A, Trsan T, Arapovic M, et al (2013) The viral chemokine MCK-2 of murine cytomegalovirus promotes infection as part of a gH/gL/MCK-2 complex. PLoS Pathog 9: e1003493. 23935483 10.1371/journal.ppat.1003493 PMC3723581

[52] Munks MW, Gold MC, Zajac AL, Doom CM, Morello CS, et al (2006) Genome-wide analysis reveals a highly diverse CD8 T cell response to murine cytomegalovirus. J Immunol 176: 3760–3766. 16517745 10.4049/jimmunol.176.6.3760

[53] Lemmermann NA, Böhm V, Holtappels R, Reddehase MJ (2011) In vivo impact of cytomegalovirus evasion of CD8 T-cell immunity: facts and thoughts based on murine models. Virus Res 157: 161–174. 20933556 10.1016/j.virusres.2010.09.022

[54] Lemmermann NA, Fink A, Podlech J, Ebert S, Wilhelmi V, et al (2012) Murine cytomegalovirus immune evasion proteins operative in the MHC class I pathway of antigen processing and presentation: state of knowledge, revisions, and questions. Med Microbiol Immunol 201: 497–512. 22961127 10.1007/s00430-012-0257-y

[55] Thimme R, Appay V, Koschella M, Panther E, Roth E, et al (2005) Increased expression of the NK cell receptor KLRG1 by virus-specific CD8 T cells during persistent antigen stimulation. J Virol 79: 12112–12116. 16140789 10.1128/JVI.79.18.12112-12116.2005 PMC1212638

[56] Obar JJ, Lefrançois L (2010) Memory CD8+ T cell differentiation. Ann NY Acad Sci 1183: 251–266. 20146720 10.1111/j.1749-6632.2009.05126.x PMC2836783

[57] Tsai M, Galli SJ (2012) IgE and mast cells in allergic disease. Nat Med 18: 693–704. 22561833 10.1038/nm.2755 PMC3597223

[58] Pennock JL, Grencis RK (2006) The mast cell and gut nematodes: damage and defence. Chem Immunol Allergy 90: 128–140. 16210907 10.1159/000088885

[59] Galli SJ, Maurer M, Lantz CS (1999) Mast cells as sentinels of innate immunity. Curr Opin Immunol 11: 53–59. 10047539 10.1016/s0952-7915(99)80010-7

[60] Echtenacher B, Männel DN, Hültner L (1996) Critical protective role of mast cells in a model of acute septic peritonitis. Nature 381: 75–77. 8609992 10.1038/381075a0

[61] Malaviya R, Ikeda T, Ross E, Abraham SN (1996) Mast cell modulation of neutrophil influx and bacterial clearance at sites of infection through TNF-α. Nature 381: 77–80. 8609993 10.1038/381077a0

[62] Stassen M, Hültner L, Schmitt E (2002) Classical and alternative pathways of mast cell activation. Crit Rev Immunol 22: 115–140. 12433130

[63] Heib V, Becker M, Taube C, Stassen M (2008) Advances in the understanding of mast cell function. Br J Haematol 142: 683–694. 18513284 10.1111/j.1365-2141.2008.07244.x

[64] St John AL, Rathore AP, Yap H, Ng ML, Metcalfe DD, et al (2011) Immune surveillance by mast cells during dengue infection promotes natural killer (NK) and NKT-cell recruitment and viral clearance. Proc Natl Acad Sci USA 108: 9190–9195. 21576486 10.1073/pnas.1105079108 PMC3107258

[65] Wang Z, Lai Y, Bernard JJ, Macleod DT, Cogen AL, et al (2012) Skin mast cells protect mice against vaccinia virus by triggering mast cell receptor S1PR2 and releasing antimicrobial peptides. J Immunol 188: 345–357. 22140255 10.4049/jimmunol.1101703 PMC3244574

[66] Aoki R, Kawamura T, Goshima F, Ogawa Y, Nakae S, et al (2013) Mast cells play a key role in host defense against herpes simplex virus infection through TNF-α and IL-6 production. J Invest Dermatol 133: 2170–2179. 23528820 10.1038/jid.2013.150

[67] Assarsson E, Kambayashi T, Sandberg JK, Hong S, Taniguchi M, et al (2000) CD8+ T cells rapidly acquire NK1.1 and NK cell-associated molecules upon stimulation in vitro and in vivo. J Immunol 165: 3673–3679. 11034371 10.4049/jimmunol.165.7.3673

[68] Kambayashi T, Assarsson E, Michaëlsson J, Berglund P, Diehl AD, et al (2000) Emergence of CD8+ T cells expressing NK cell receptors in influenza A virus-infected mice. J Immunol 165: 4964–4969. 11046023 10.4049/jimmunol.165.9.4964

[69] Redwood AJ, Shellam GR, Smith LM (2013) Molecular evolution of murine cytomegalovirus genomes. In: Reddehase MJ, editor. Cytomegaloviruses: from molecular pathogenesis to intervention. Caister Academic Press, Norfolk, UK. Vol. I. pp 23–37

[70] Galli SJ, Kalesnikoff J, Grimbaldeston MA, Piliponsky AM, Williams CM, et al (2005) Mast cells as “tunable” effector and immunoregulatory cells: recent advances. Annu Rev Immunol 23: 749–786. 15771585 10.1146/annurev.immunol.21.120601.141025

[71] Wagner M, Jonjic S, Koszinowski UH, Messerle M (1999) Systematic excision of vector sequences from the BAC-cloned herpesvirus genome during virus reconstitution. J Virol 73: 7056–7060. 10400809 10.1128/jvi.73.8.7056-7060.1999 PMC112796

[72] Podlech J, Holtappels R, Grzimek NK, Reddehase MJ (2002) Animal models: murine cytomegalovirus. In: Kaufmann SHE, Kabelitz D, editors. Methods in Microbiology: Immunology of Infection. 2^nd^ ed. Academic Press, London. Pp 493–525.

[73] Cobbold SP, Jayasuriya A, Nash A, Prospero TD, Waldmann H (1984) Therapy with monoclonal antibodies by elimination of T-cell subsets in vivo. Nature 312: 548–551. 6150440 10.1038/312548a0

[74] Jonjic S, Pavic I, Lucin P, Rukavina D, Koszinowski UH (1990) Efficacious control of cytomegalovirus infection after long-term depletion of CD8+ T lymphocytes. J Virol 64: 5457–5464. 1976821 10.1128/jvi.64.11.5457-5464.1990 PMC248597

[75] Stassen M, Valeva A, Walev I, Schmitt E (2006) Activation of mast cells by streptolysin O and lipopolysaccharide. Methods Mol Biol 315: 393–403. 16110172 10.1385/1-59259-967-2:393

[76] Reuter S, Heinz A, Sieren M, Wiewrodt R, Gelfand EW, et al (2008) Mast cell-derived tumour necrosis factor is essential for allergic airway disease. Eur Respir J 31: 773–782. 18094004 10.1183/09031936.00058907

[77] Reuter S, Dehzad N, Martin H, Heinz A, Castor T, et al (2010) Mast cells induce migration of dendritic cells in a murine model of acute allergic airway disease. Int Arch Allergy Immunol 151: 214–222. 19786802 10.1159/000242359

[78] Maxeiner JH, Karwot R, Hausding M, Sauer KA, Scholtes P, et al (2007) A method to enable the investigation of murine bronchial immune cells, their cytokines and mediators. Nat Protoc 2: 105–112. 17401344 10.1038/nprot.2007.8

[79] Pahl-Seibert MF, Juelch M, Podlech J, Thomas D, Deegen P, et al (2005) Highly protective in vivo function of cytomegalovirus IE1 epitope-specific memory CD8 T cells purified by T-cell receptor-based cell sorting. J Virol 79: 5400–5413. 15827154 10.1128/JVI.79.9.5400-5413.2005 PMC1082747

[80] Böhm V, Simon CO, Podlech J, Seckert CK, Gendig D, et al (2008) The immune evasion paradox: immunoevasins of murine cytomegalovirus enhance priming of CD8 T cells by preventing negative feedback regulation. J Virol 82: 11637–11650. 18815306 10.1128/JVI.01510-08 PMC2583666

[81] Lemmermann NA, Podlech J, Seckert CK, Kropp KA, Grzimek NK, et al. (2010) CD8 T-cell immunotherapy of cytomegalovirus disease in the murine model. In: Kabelitz D, Kaufmann SHE, editors. Methods in Microbiology: Immunology of Infection. 3^rd^ ed. Academic Press, London. pp 369–420.

