# Supplementary figures and images for: Mast Cells Expedite Control of Pulmonary Murine Cytomegalovirus Infection by Enhancing the Recruitment of Protective CD8 T Cells to the Lungs
Source: PLoS Pathog. 2014 Apr 24;10(4):e1004100. doi: 10.1371/journal.ppat.1004100 (PMC3999167; doi:10.1371/journal.ppat.1004100)

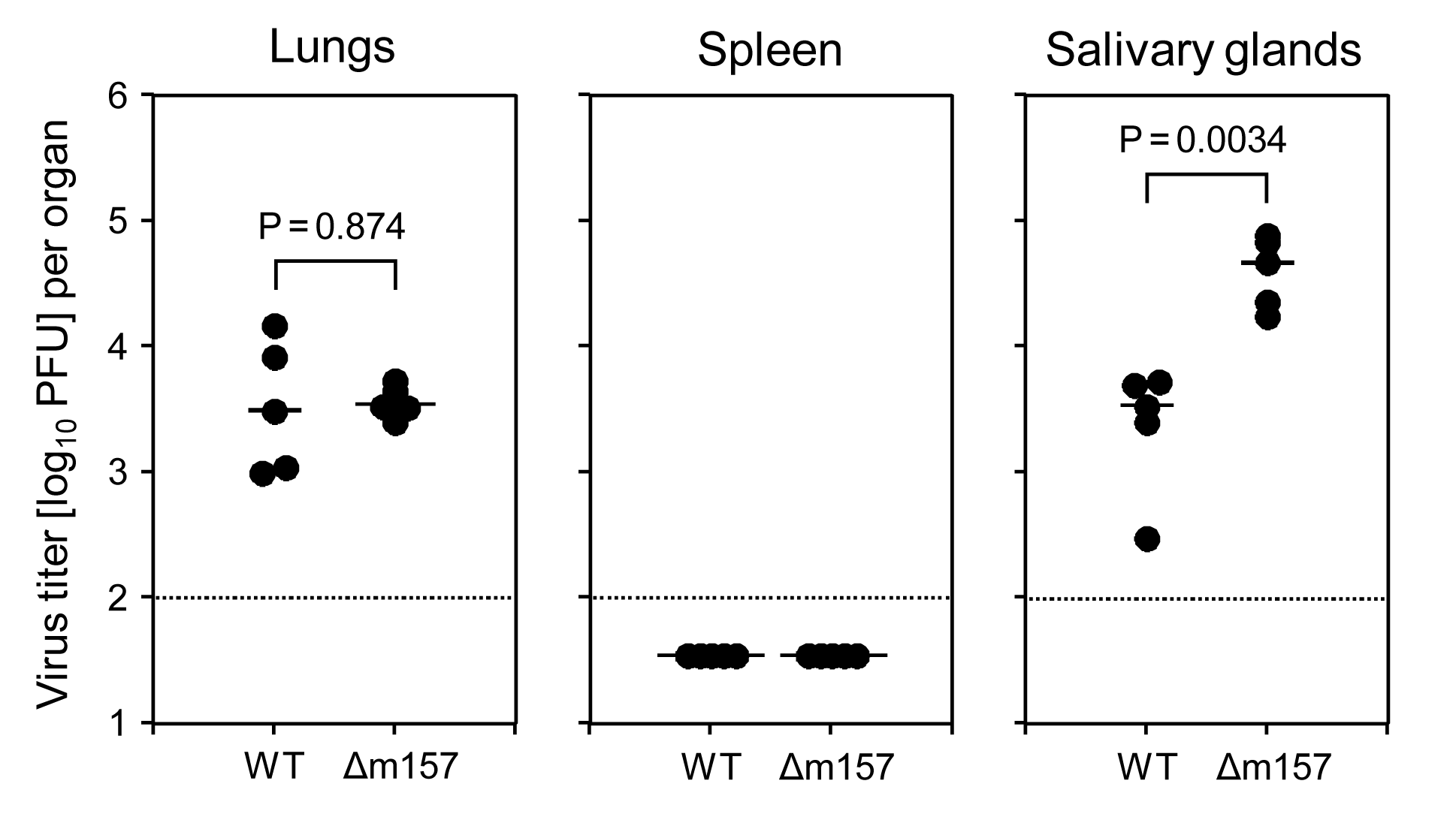

Supplement: Figure S1 — Ly49H+ NK cells stimulated by viral ligand m157 are not involved in the control of pulmonary infection at a later stage. C57BL/6 mice were infected either with WT virus or with mutant virus mCMV-Δm157 lacking the viral gene m157 that encodes a potent ligand of the activatory NK cell receptor Ly49H. Virus titers in the indicated organs were determined on day 14 post-infection. Dots represent virus titers in individual mice revealing the sample sizes and ranges, with the median values indicated. The dotted lines indicate the detection limits. (TIF) [file ppat.1004100.s001.tif]

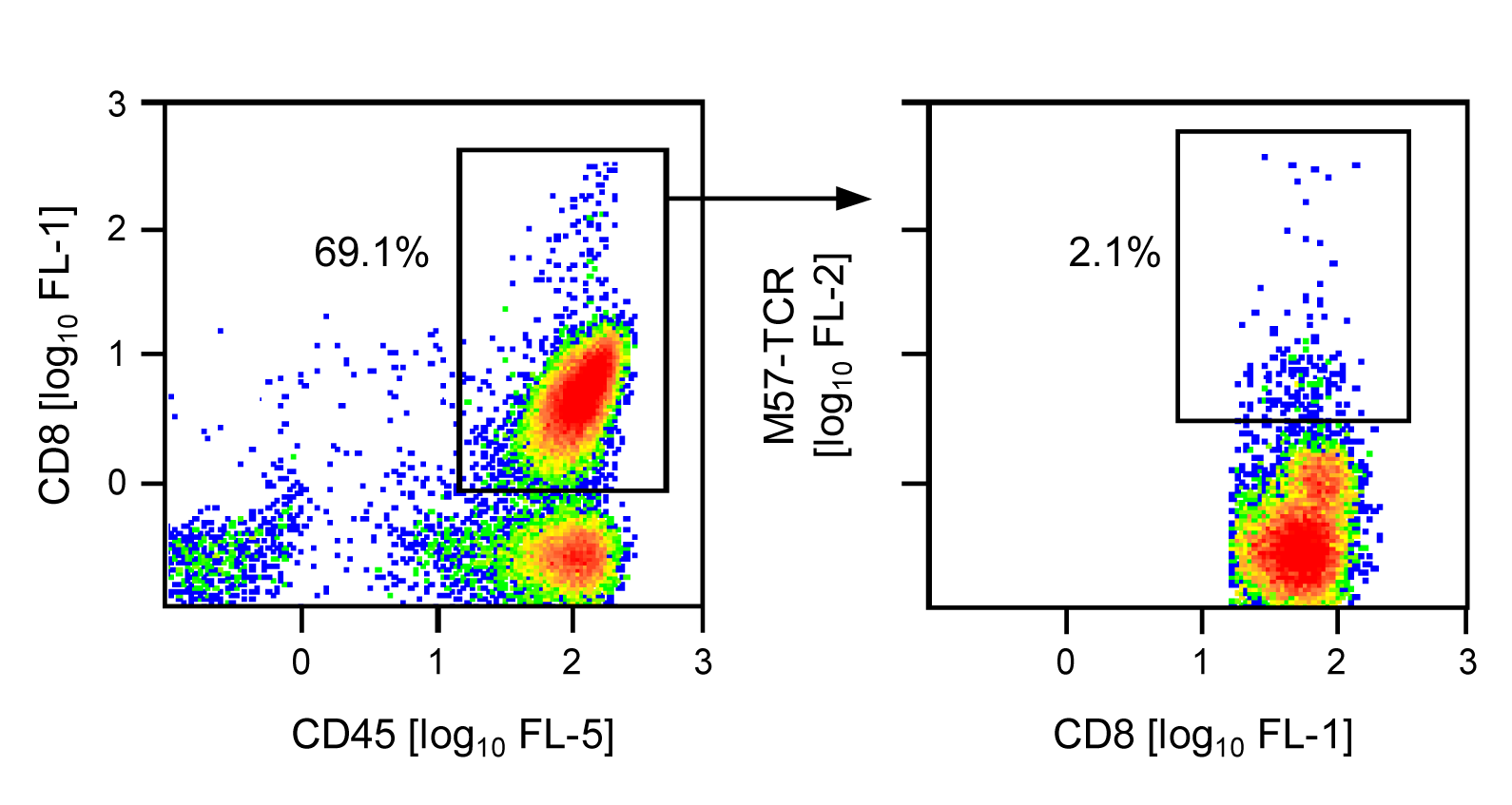

Supplement: Figure S2 — Cytofluorometric quantitation of viral epitope-specific CD8 T cells recovered by BAL. Corresponding to the data shown in Figure 2, leucocytes retrieved by BAL were analyzed cytofluorometrically. A gate was set on CD45+CD8+ T cells, and within this population, cells carrying a TCR specific for the M57 epitope (M57-TCR) were identified by binding of fluorochrome-conjugated peptide-MHC class-I (SCLEFWQRV-H-2Kb) multimer. Shown are color-coded density plots with red and blue representing highest and lowest density, respectively. (FL) fluorescence channel and fluorescence intensity. Percentages of main interest are indicated for gated areas. (TIF) [file ppat.1004100.s002.tif]

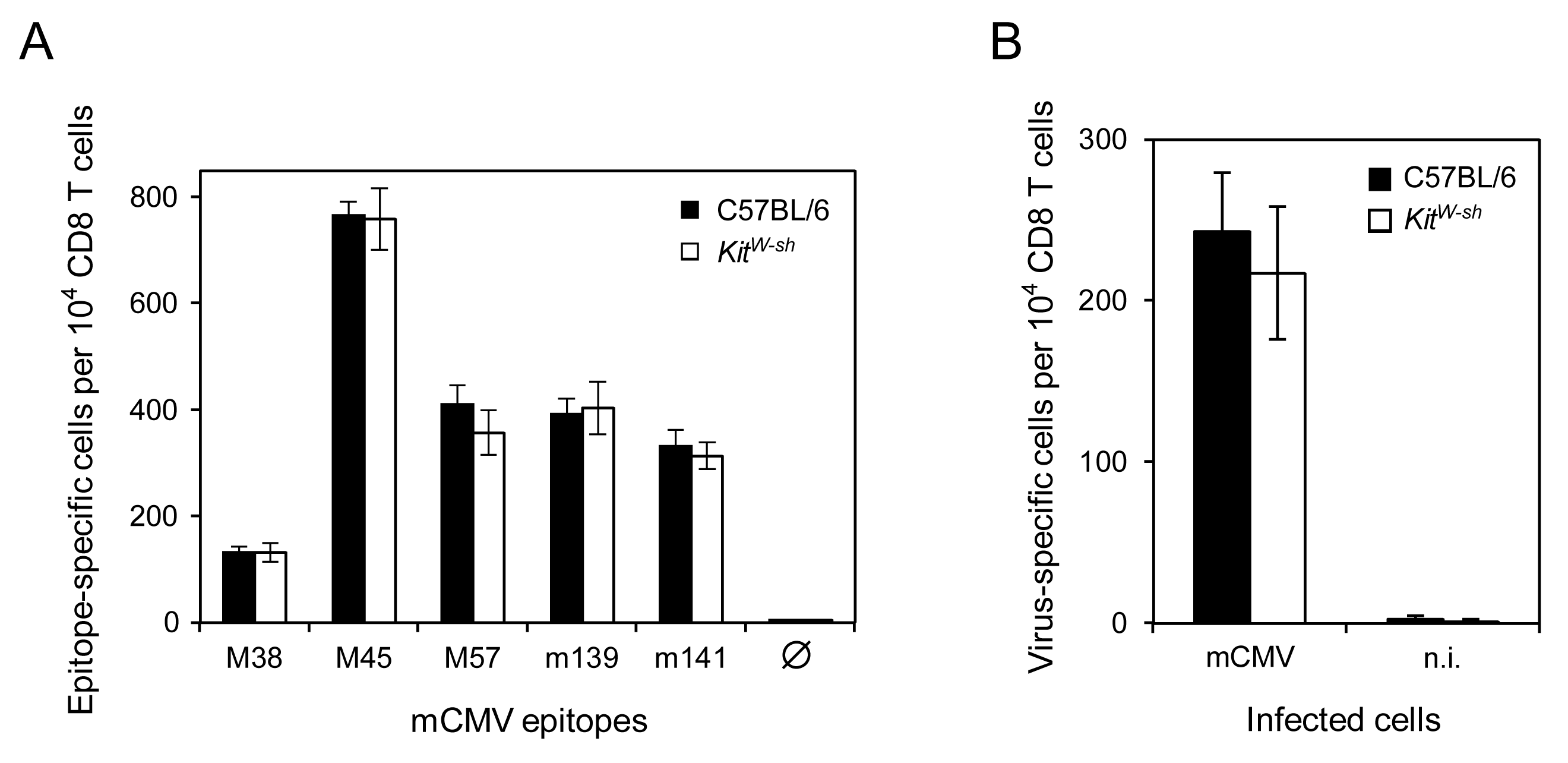

Supplement: Figure S3 — MC deficiency has no influence on the viral epitope hierarchy of the CD8 T-cell response in the lungs nor on recognition of infected cells. MC-sufficient WT C57BL/6 (black bars) and MC-deficient KitW-sh (open bars) mice were infected intravenously. On day 6 post-infection, immunomagnetically-purified CD8 T lymphocytes derived from the lungs of 6 mice per group were used as responder cells in IFNγ-based ELISpot assays. (A) Hierarchy of viral epitopes recognized by pulmonary CD8 T cells. EL-4 stimulator cells were exogenously loaded with synthetic peptides representing the indicated MHC-I-presented epitopes. (Ø) stimulator cells with no peptide added. (B) Recognition of infected cells (MEF) presenting naturally-processed viral peptides despite the expression of viral immune evasion genes. (n.i.) uninfected MEF. Throughout, frequencies of responding, IFNγ-secreting cells were determined by intercept-free linear regression analysis. Bars represent most probable numbers, and error bars indicate 95% confidence intervals. (TIF) [file ppat.1004100.s003.tif]

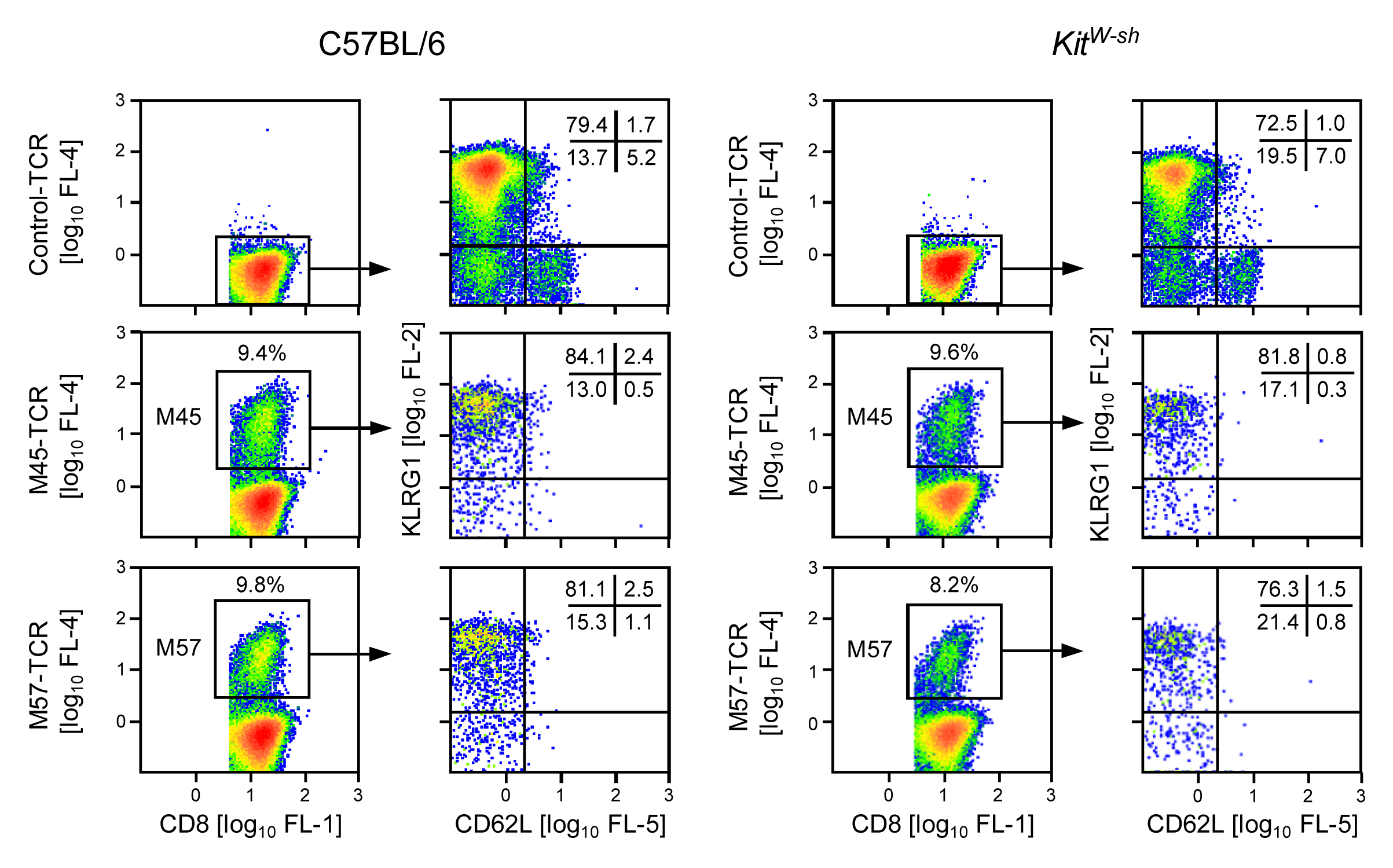

Supplement: Figure S4 — MC deficiency does not notably alter the activation phenotype of pulmonary CD8 T cells. MC-sufficient WT C57BL/6 (left panels) and MC-deficient KitW-sh (right panels) mice were infected intravenously. Multi-color cytofluorometric analyses were performed on day 6 post-infection for lung infiltrate cells pooled from 6 mice per group and pre-gated on CD8+ cells (for the gating strategy, recall Fig. 1B). Shown are color-coded density plots with red and blue representing highest and lowest density, respectively. The analyzed cell surface markers are indicated. Viral epitope-specific CD8 T cells were identified with TCR-specific peptide-MHC class-I multimers, specifically with M45-Db and M57-Kb Dextramers. (FL) fluorescence channel and fluorescence intensity. Percentages of main interest are indicated for gated areas and quadrants. (TIF) [file ppat.1004100.s004.tif]

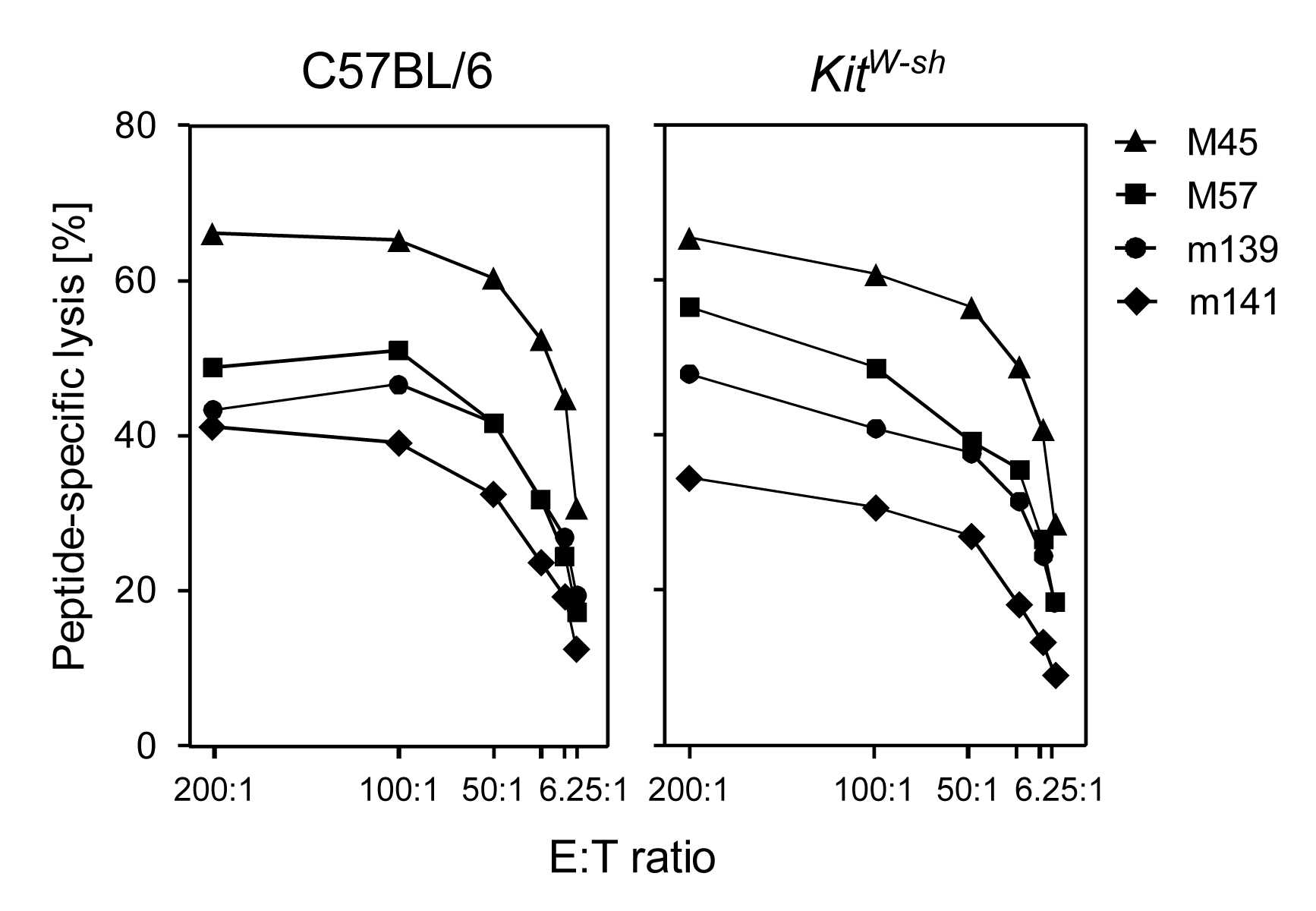

Supplement: Figure S5 — MC deficiency does not alter the ex vivo cytolytic activity of pulmonary CD8 T cells. Immunomagnetically-purified CD8 T cells from infected MC-sufficient WT C57BL/6 and MC-deficient KitW-sh mice were derived from the experiment described in Figure S3, so that the different effector functions can be directly compared. Here, the CD8 T cells were analyzed ex vivo for their cytolytic effector function with no preceding expansion in cell culture. Viral epitope-specific cytolysis was assayed at graded effector-to-target (E∶T) cell ratios with EL-4 lymphoma cells as target cells pulsed with saturating concentrations of synthetic peptides corresponding to the viral epitopes indicated. (TIF) [file ppat.1004100.s005.tif]
